# Information-Content-Informed Kendall-Tau Correlation Methodology: Interpreting Missing Values in Metabolomics as Potentially Useful Information

**DOI:** 10.3390/metabo16040245

**Published:** 2026-04-04

**Authors:** Robert M. Flight, Praneeth S. Bhatt, Hunter N. B. Moseley

**Affiliations:** 1 Markey Cancer Center, University of Kentucky, Lexington, KY 40536, USA; robert.flight@uky.edu; 2 Department of Molecular & Cellular Biochemistry, University of Kentucky, Lexington, KY 40536, USA; 3 Superfund Research Center, University of Kentucky, Lexington, KY 40536, USA; 4 Department of Electrical and Computer Engineering, University of Kentucky, Lexington, KY 40506, USA; praneethsbhatt@gmail.com; 5 Institute for Biomedical Informatics, University of Kentucky, Lexington, KY 40536, USA; 6 Department of Toxicology and Cancer Biology, University of Kentucky, Lexington, KY 40536, USA

**Keywords:** metabolomics, correlation, missingness, left-censored

## Abstract

**Background:** Almost all correlation measures currently available are unable to directly handle missing values. Typically, missing values are either ignored completely by removing them or are imputed and used in the calculation of the correlation coefficient. In either case, the correlation value will be impacted based on the perspective that the missing data represents no useful information. However, missing values occur in real datasets for a variety of reasons. In metabolomics datasets a major reason for missing values is that a specific measurable phenomenon falls below the detection limits of the analytical instrumentation (left-censored values). These missing data are not missing at random, but represent potentially useful information by virtue of their “missingness” at one end of the data distribution. **Methods:** To include this information due to left-censored missingness, we propose the information-content-informed Kendall-tau (ICI-Kt) methodology. We develop a statistical test and then show that most missing values in metabolomics datasets are the result of left-censorship. Next, we show how left-censored missing values can be included within the definition of the Kendall-tau correlation coefficient, and how that inclusion leads to an interpretation of information being added to the correlation. We also implement calculations for additional measures of theoretical maxima and pairwise completeness that add further layers of information interpretation in the methodology. **Results:** Using both simulated and over 700 experimental data sets from the Metabolomics Workbench, we demonstrate that the ICI-Kt methodology allows for the inclusion of left-censored missing data values as interpretable information, enabling both improved determination of outlier samples and improved feature–feature network construction. **Conclusions:** We provide explicitly parallel implementations in both R and Python that allow fast calculations of all the variables used when applying the ICI-Kt methodology on large numbers of samples. The ICI-Kt methods are available as an R package and Python module on GitHub.

## 1. Introduction

Correlation as a measure of the relatedness or similarity of two or more sets of data has a long history, with the mathematical technique being used (and abused) in various scientific fields since its introduction [1,2]. More recently, correlation calculations have become a cornerstone statistical method in the analysis and integration of varied omics datasets, especially the big five omics: genomics, transcriptomics, proteomics, metabolomics, and epigenomics [3]. Correlation between biomolecular features (nucleotide variants, RNA transcripts, proteins, metabolites, DNA methylation, etc.) may be used to evaluate the relationship strength between pairs of the features, as well as to detect and derive correlative structures between groups of features [4]. Moreover, feature–feature correlations can be used to evaluate a dataset based on expected biochemical correlations, for example, higher feature–feature correlations within lipid categories versus between lipid categories [5]. Correlation is a foundational method for generating biomolecular feature–feature interaction networks, like those provided by STRING [6], Genemania [7], and WCGNA [8]. Feature–feature correlation may also be used to inform which features are used for the imputation of missing values [9].

Often, the first step in omics-level analyses is to examine the sample–sample (dis)similarities in various ways using exploratory data analysis or EDA. This can include the examination of decomposition by principal components analysis (PCA), sample–sample pairwise distances, or sample–sample pairwise correlations to highlight biological and batch groups [10,11,12], double-check the appropriateness of planned analyses [13], and check if any samples should be removed prior to statistical analysis (outlier detection and removal) [14]. Outlier detection, in particular, is often required for successful omics data analysis, as any misstep during the experimentation, sample collection, sample preparation, or analytical measurement of individual samples can inject high error and/or variance into the resulting dataset [10,11,12,14,15].

**Figure 1:**
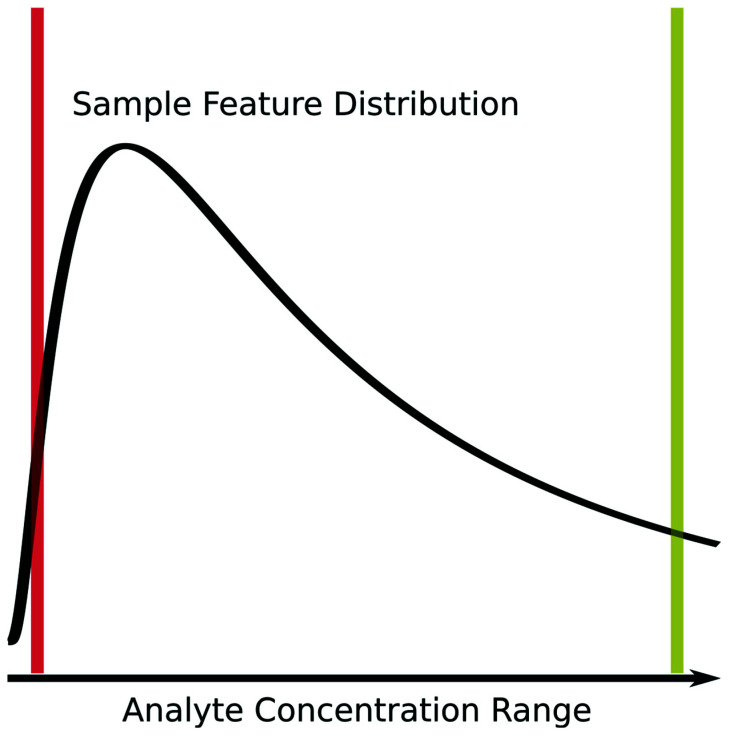
Graphical description of the left-censored data problem. An example density plot of the analyte concentrations for a single experimental sample is shown as a solid black curve. The true analyte concentration range covers the full range of the density distribution, with the minimum on the left (red vertical line) and the maximum on the right (yellow vertical line). Below certain concentrations, shown by the red line, the instrument returns either missing values (NA), zeros, or some other floored values, resulting in a left-censored distribution. Above certain concentrations, highlighted by the yellow line, typically the values returned will be identical (or flagged depending on the instrument). Which analytes will have concentrations below the red detection limit line may vary from sample to sample due to the overall sample composition, as well as biological variance.

All analytical methods, and, in particular, the analytical methods used in omics, where many analytes are being measured simultaneously, suffer from missing measurements. Some analytes will be missing at random because of spurious issues with either the instrument, the particular sample, or sample preparation, but a larger number of missing measurements are left-censored due to analytes being below the effective detection limit of the instrument and the given specific sample preparation procedures utilized, as shown in Figure 1. Some analytical instruments are purposely designed to floor measurements when they occur below a certain signal-to-noise-ratio threshold. Also, imputation of missing measurements in omics samples is an active area of research, which we will not comprehensively cover here, beyond saying that it is worthwhile and very necessary in many instances. Imputation methods rely on very similar analytical detection limits between analytical samples. When this condition does not hold, imputation methods have reduced performance and lower interpretive value. For analytical techniques requiring complex sample handling and detection, the variability in the analytical detection level can be quite high. Some differential analysis methods have been developed to directly handle missing values in statistical testing methodology [16,17]. However, when it comes to calculating correlation, there are very few methods that explicitly account for left-censored missing data that we know of. In many cases, missing values are either ignored or imputed to zero (or another value) and then included in the correlation calculation. The two most common approaches for ignoring (i.e., dropping) values are to only use those measurements that are common across all samples (complete) or that are common between two samples being compared (pairwise-complete). Both dropping and imputing missing values are likely to cause the calculated sample–sample correlation values to deviate from the real sample–sample correlation values, especially with respect to specific data interpretation perspectives.

Assuming that a majority of missing values are not missing at random, but rather result from left-censored distributions due to the analyte being below the effective detection limit (see Figure 1), we propose that these missing values do in fact encode useful information that can be incorporated into correlation calculations. Thus, information content, i.e., the amount of information available and used, can be either increased or at least not lost in the calculation of the correlation.

To create a correlation measure that is capable of working with missing values, we are not interested in creating a completely new correlation metric from scratch, but in modifying an existing one. Of the three commonly used correlation measures, Pearson, Spearman, and Kendall-τ, Spearman and Kendall-τ seem most appropriate for modification as they solely use ranks in the calculation of their coefficients. Modifying Pearson would either involve imputing new values or finding a way to calculate the covariances with missingness included. While Spearman uses ranks, many of the modifications for handling identical ranks and ties do not seem amenable to working with missing values. In contrast, Kendall-τ’s use of concordant- and discordant-pair counts seems most amenable to the creation of new definitions that incorporate missingness while still working within the original definition of the correlation coefficient, as shown in Section 2.6 below.

In this work, we propose new definitions of concordant and discordant rank pairs that include missing values, as well as methods for incorporating missing values into the number of tied values for the equivalent of the modified Kendall-τ coefficient, the information-content-informed Kendall-τ (ICI-Kt) method. The implementation of the basic calculation of ICI-Kt involves the replacement of missing values with a value lower than the observed values (technically simple imputation), with subsequent calculation of the Kendall $\tau_{b}$ statistic; as a majority of missing values are the result of left-censorship, they still provide an interpretation from an information content perspective, which we demonstrate with the equations below. We also developed a binomial statistical test for determining if the cause for missingness is likely left-censorship. With this statistical test and experimental datasets from the Metabolomics Workbench (MW) [18], we demonstrate that left-censorship is the cause of many missing values across a large number of metabolomics datasets. We examine the effect of missing values on various collections of simulated and real datasets, comparing the ICI-Kt methodology with other simpler methods of handling the missing values, namely removing them or imputing them to zero. Given the detrimental effects of including outlier samples, we also evaluate the application of ICI-Kt in quality control and quality assessment steps typically performed prior to differential analyses. Specifically, we compare ICI-Kt to other common correlation-based outlier detection methods. We were also curious about the utility of the ICI-Kt methodology in creating metabolomics feature–feature networks with large amounts of missing values, so we evaluated the partitioning of networks by Reactome pathways [19] after network creation using different correlation measures.

All of the code and data used for this manuscript is available on Zenodo [20].

## 2. Materials and Methods

### 2.1. Additional Definitions of Concordant and Discordant Pairs to Include Missingness

In the simplest form, the Kendall-τ ($\tau_{a}$) correlation can be defined as follows:

$$\tau_{a}=\frac{n_{concordant}-n_{discordant}}{n_{concordant}+n_{discordant}}$$

where $n_{concordant}$ is the number of concordant pairs and $n_{discordant}$ is the number of discordant pairs. In this case, a pair is any two x-y points, $x_{i},y_{i}$ and $x_{j},y_{j}$, with i≠j, composed from two jointly random variables X and Y, where $x_{i}$ represents the *ith* value in X and $y_{i}$ represents the *ith* value in Y. In a metabolomics context, X and Y can represent metabolite feature vectors for two experimental samples or two specific metabolite features across a set of samples.

A concordant pair has the following classical definition:

- $x_{i}>x_{j}$ and $y_{i}>y_{j}$
- $x_{i}<x_{j}$ and $y_{i}<y_{j}$

A discordant pair has the following classical definition [21]:

- $x_{i}>x_{j}$ and $y_{i}<y_{j}$
- $x_{i}<x_{j}$ and $y_{i}>y_{j}$

We can expand the concordant- and discordant-pair definitions to include missing values (e.g., NA in R). The information-content-informed concordant-pair definitions are then as follows:

- $x_{i}>x_{j}$ and $y_{i}>y_{j}$
- $x_{i}<x_{j}$ and $y_{i}<y_{j}$
- $x_{i}>x_{j}$ and $y_{i}\neq \mathrm{NA},\;\;y_{j}=\mathrm{NA}$
- $x_{i}<x_{j}$ and $y_{i}=\mathrm{NA},\;\;y_{j}\neq \mathrm{NA}$
- $x_{i}\neq \mathrm{NA},\;\;x_{j}=\mathrm{NA}$ and $y_{i}>y_{j}$
- $x_{i}=\mathrm{NA},\;\;x_{j}\neq \mathrm{NA}$ and $y_{i}<y_{j}$
- $x_{i}\neq \mathrm{NA},\;\;x_{j}=\mathrm{NA}$ and $y_{i}\neq \mathrm{NA},\;\;y_{j}=\mathrm{NA}$
- $x_{i}=\mathrm{NA},\;\;x_{j}\neq \mathrm{NA}$ and $y_{i}=\mathrm{NA},\;\;y_{j}\neq \mathrm{NA}$

The information-content-informed discordant-pair definitions are then as follows:

- $x_{i}>x_{j}$ and $y_{i}<y_{j}$
- $x_{i}<x_{j}$ and $y_{i}>y_{j}$
- $x_{i}>x_{j}$ and $y_{i}=\mathrm{NA},\;\;y_{j}\neq \mathrm{NA}$
- $x_{i}<x_{j}$ and $y_{i}\neq \mathrm{NA},\;\;y_{j}=\mathrm{NA}$
- $x_{i}\neq \mathrm{NA},\;\;x_{j}=\mathrm{NA}$ and $y_{i}<y_{j}$
- $x_{i}=\mathrm{NA},\;\;x_{j}\neq \mathrm{NA}$ and $y_{i}>y_{j}$
- $x_{i}\neq \mathrm{NA},\;\;x_{j}=\mathrm{NA}$ and $y_{i}=\mathrm{NA},y_{j}\neq \mathrm{NA}$
- $x_{i}=\mathrm{NA},\;\;x_{j}\neq \mathrm{NA}$ and $y_{i}\neq \mathrm{NA},y_{j}=\mathrm{NA}$

These additional definitions make it possible to interpret a Kendall-τ correlation from the perspective of missing values as additional information, i.e., information-content-informed Kendall-τ (ICI-Kt) methodology.

### 2.2. Considering Ties

Tied values do not contribute to either of the concordant- or discordant-pair counts, and the original Kendall-τ formula for the $\tau_{a}$ statistic does not consider the presence of tied values. However, the related $\tau_{b}$ statistic does handle the presence of tied values by adding the tied x and y values to the denominator, and in our special case of missing data, we can add the ties that result from $\left(x_{i}=\mathrm{NA},\;x_{j}=\mathrm{NA}\right)$ and $\left(y_{i}=\mathrm{NA},\;y_{j}=\mathrm{NA}\right)$ to $n_{xtie}$ and $n_{ytie}$ [22,23] used in the following equation for $\tau_{b}$:

$$\tau_{b}=\frac{n_{concordant}-n_{discordant}}{\sqrt{\left(n_{tot}-n_{xtie}\right)\left(n_{tot}-n_{ytie}\right)}}$$

where $n_{tot}$ is the total number of pairs, $n_{xtie}$ is the number of tied values in X, and $n_{ytie}$ is the number of paired values in Y.

We can also consider commonly missing values in X and Y specially as well. In the first instance, we remove those x-y points where both values are missing, preventing their interpretation as missing information content. We refer to this case as the *local* ICI-Kt correlation. It is most appropriate for the comparison of only two experimental samples, where we are concerned with what values are present in the two experimental samples, with the odd case of missingness.

The other case, where we leave ties resulting from points with missing X and Y, we refer to as the *global* ICI-Kt correlation. In this case, every single correlation over multiple comparisons with the same set of metabolite features will consider the same number of pair comparisons. This is useful when analyzing and interpreting correlations from a large number of experimental samples, not just two samples, since x-y points where both values are missing are interpreted as missing information content.

### 2.3. p-Value

With the calculation of the number of entries, and the numbers of ties (which may change depending on whether one is using the global or local correlations), a *p*-value for the correlation can be calculated using the Mann–Kendall test [24].

### 2.4. Theoretical Maxima

The *global* case also provides an interesting property, whereby we can calculate the theoretical maximum correlation that would be possible to observe given the lowest number of shared missing values. This value can be useful to scale the rest of the observed correlation values across many sample–sample correlations, providing a value that scales an entire dataset appropriately. For any pairwise comparison of two vectors (from experimental samples, for example), we can calculate the maximum possible Kendall-tau for that comparison by defining the maximum number of concordant pairs as follows:

$$\tau_{max}=\frac{n_{tot}-n_{xtie}-n_{ytie}+n_{tie}}{\sqrt{\left(n_{tot}-n_{xtie}\right)\left(n_{tot}-n_{ytie}\right)}}$$

where $n_{tie}$ is the number of commonly tied values in both X and Y. Calculating a set of $\tau_{max}$ values between all experimental samples, we can take the maximum of the values, and use it to scale all of the obtained Kendall-tau values equally ($\frac{\tau }{max\left(\tau_{max}\right)}$).

We do note that scaling by $max\left(\tau_{max}\right)$ changes the overall values returned from a particular dataset, and makes comparisons between datasets invalid. Therefore, when calculating ICI-Kt values for comparison between datasets, we advise users to set the scale_max option to FALSE.

### 2.5. Completeness

As an addition to the correlation value, we also calculate the *completeness* between any two samples. We first measure the number of entries missing in either of the samples being compared, and subtract that from the total number of features in the samples. This defines how many features are potentially *complete* between the two samples. This number, over the total number of features, defines the *completeness* fraction.

$$\mathrm{completness}=\frac{n_{feat}-N\left(miss_{i}\cup miss_{j}\right)}{n_{feat}}$$

where for any two samples *i* and *j*, $n_{feat}$ is the total number of features or entries, and $miss_{i}\cup miss_{j}$ are the metabolite features missing in either sample *i* or *j*, with N being the total number of missing entries in either sample *i* or *j*.

### 2.6. Implementation Details

We produced an initial reference implementation in base R [25], where the various concordant- and discordant-pair definitions were written as simple logical tests to allow further exploration and validation of faster implementations. During exploration and validation of an early implementation, we discovered that an equivalent calculation was to replace the missing values with a value smaller than all of the values in the two sets being compared. This simplification does not change the interpretation of the effect of left-censored missing values, but it does allow for the direct use of the very fast mergesort-based algorithm for calculating $\tau_{b}$ [26].

We re-implemented the mergesort implementation from the SciPy kendalltau code [27] in both R (via Rcpp) and Python (via Cython) to enable fast, easy parallel computations in both languages (using furrr and multiprocessing, respectively), as well as the inclusion of the calculation of $tau_{max}$, which is derived from the same values needed for the calculation of $\tau_{b}$ (see above). For consistency, we also re-implemented the calculation of the *p*-values for the $\tau_{b}$ statistic from the SciPy implementation, which follows the description of the Mann–Kendall test [24]. The version of the ICIKendallTau R package used in this manuscript is available on Zenodo [28]. In addition to use as an imported Python module, the Python icikt module provides a command line interface (CLI) for the ICI-Kt methodology.

### 2.7. Simulated Datasets

Distribution parameters for simulated datasets are listed in Table 1. Simulated feature vectors (analytical samples) are generated by drawing random values from a log-normal distribution and sorting them in ascending order to create a pair of samples with perfectly positive (1) or negative (−1) correlation values (perfect dataset). Log-normal distributions were used for the initial distribution as our experience with analyzing several mass-spectrometry datasets has shown they frequently follow a log-like distribution, and it is necessary to log-transform the data prior to further analysis. This is also supported by Abram and McCloskey [29]. Random variance is added to one of the two samples by drawing values from a uniform distribution over −0.5 to 0.5, and adding the values to the original sample, and sorting them again to maintain a correlation of 1 or −1 for Kendall-τ correlation (noise-1). A sample with a small percentage (0.5%) of outlier points at one end of the distribution is created by sampling from a uniform distribution over the range −0.5 to 0.5, and then a log-normal distribution (outlier dataset), and adding the log-normal values to the uniform values for a combined source of random variance that is added to the original sample values. The negative analytical sample has values sorted in descending order. Missing value indices are generated by randomly sampling up to 499 of the lowest values in each sample. For the negative sample, the indices are also subtracted from 1000 to cause them to be at the lower end of the feature distribution. Finally, missing indices were only inserted into one of the two samples being compared before calculating the correlation. The missing indices are replaced with NA, and then correlations between the analytical samples are calculated.

Another, more realistic, simulated dataset is generated by drawing from a log-normal distribution, and adding noise from a normal distribution to create two statistical samples (realistic dataset and noise-2). Missing values are created in these statistical samples via two methods: (1) by creating intensity cutoffs from 0 to 1.5 in 0.1 increments, values below the cutoff are set to missing or zero depending on the calculation; (2) randomly sampling locations in the two-sample matrix ranging from zero to 300 in increments of 50 and setting the indices to missing or zero.

### 2.8. Metabolomics Datasets from Metabolomics Workbench

A set of 6105 analysis datasets from the Metabolomics Workbench (MW) were downloaded on 12 November 2025 using the mwtab Python package [30], and repaired to fix various issues. For a subset that had metabolite feature abundances outside the mwtab json file, the files were downloaded on 13 November 2025. The various pieces of each dataset were parsed and transformed into R-appropriate structures, mainly data frames of various types for metadata, and matrices of abundances (see Data Processing). Subject sample factors (SSFs) were transformed so that each sample had a combination of various factors to describe the unique groups of samples. For example, if a dataset included samples with one or more genotypes (Knockout, FLOX) and taken from different segments of the intestines, the final factor for each sample is the combination of genotype + intestinal segment.

For inclusion in this work, an analysis dataset had to meet these criteria:

- ≥100 metabolites, so that any degree of missingness would still allow for robust estimation of correlations between samples.
- One SSF grouping with ≥5 samples, and ≥2 SSF groupings after removing samples that may be pooled, quality control or blanks; this provides a greater likelihood of decent variance estimates when calculating the F-statistics across SSFs after removing potential outlier samples.
- A maximum metabolite feature abundance ≥ 20 to exclude log-transformed values and low-dynamic-range datasets.
- The ability to calculate a correlation between the median rank of a metabolite feature and the number of samples the metabolite was missing within a factor, as this indicated a minimum number of missing values in each SSF.

Of the 6105 datasets initially downloaded, 711 were kept for further analysis.

### 2.9. Number of Missing Values and Median Rank

For each dataset, the samples were split by SSF (see previous Methods). For each metabolite feature, the rank of the feature was calculated for each sample where the feature was present, followed by the feature’s median rank across samples, as well as the number of samples the feature was missing from. Grouping the features by the number of missing values, we calculate the median of median ranks, as well as the minimum of median ranks, for the visualization and correlation of the relationship of rank with missing values.

### 2.10. Binomial Test for Left-Censorship

For each dataset, the samples are first split by SSF. In each sample, the median abundance of features present in the sample is calculated. For any feature that is missing in any sample, the values in the present samples are compared to the median value of their corresponding sample. If the value is less than or equal to the median value in the sample, that is counted as a success in a binomial test; otherwise, it is counted as a failure. The number of successes and failures is aggregated across the SSF splits for calculation in a binomial test, with the null hypothesis assuming a ratio of 0.5.

### 2.11. Correlation Methods

For each dataset, we calculated correlations using a variety of methods. Across the various datasets, either zeros or empty strings (generally resulting in NA values when read into R) are used to represent missingness. To start, we replaced all missing values with NA, and then either left them as NA or set them to zero for the various methods used: ICI-Kt with NA (icikt), and then scaled (multiplied) by the completeness metric (icikt_complete); Kendall-tau, with NA, and then using pairwise-complete-observations (kt_base); Pearson, with NAs replaced by zeros, using pairwise-complete-observations (pearson_base); Pearson, with NA, using pairwise-complete-observations (pearson_base_nozero); Pearson, with a $log\left(x+1\right)$ transform applied, using pairwise-complete-observations (pearson_log1p); and Pearson, with a $log\left(x\right)$ transform, and then setting infinite values to NA values, using pairwise-complete-observations (pearson_log).

### 2.12. Outlier Detection

For outlier detection, median sample–sample correlations within the unique SSF (genotype, condition, and their combinations) is calculated, and $log\left(1-cor_{median}\right)$ is calculated to transform it into a score. Then, outliers are determined using R’s grDevices::boxplot.stats, which by default are at 1.5X the whiskers in a box-and-whisker plot. This is functionally equivalent to determining outliers as values that are ≥1.5X the interquartile range of the data. As we are interested in only those correlations at the low end of correlation (becoming the high end after the subtraction and log-transform), we restrict to only those entries at the high end of the score distribution (using visualizationQualityControl::determine_outliers [31]). This is equivalent to using the correlation component of the score described by Gierliński et al. [14] and setting the other component weights to zero.

After outlier detection, the ANOVA statistics are calculated across SSFs using limma (v 3.62.2) [32], and the fraction of metabolites with an adjusted *p*-value ≤0.05 is recorded (Benjamini–Hochberg adjustment [33]). The correlation methods are compared by their fractions of significant metabolites, using a paired *t*-test and adjusting *p*-values across method comparisons using the Bonferroni adjustment.

### 2.13. Feature Annotations

Predicted Reactome pathway annotations for analyzable MW datasets were parsed from the previous work by Huckvale et al. [19,34]. Given the hierarchical nature of Reactome pathways, and the use of the pathways for partitioning feature–feature networks, we aggregated the Reactome pathways into larger grouped pathway sets with less feature overlap. First, we aggregated predicted pathway annotations to the same pathway identifier across species. Second, for each pathway annotation with ≥20 and ≤500 features, we calculated the combined overlap metric between all pathways (categoryCompare2 v 0.200.4 [35,36], values range between 0 and 1). Treating the overlap metric between pathways as weighted edges of a graph, we removed the edges with a value ≤0.6, and then did community detection using the walktrap clustering method in the igraph package (v 2.1.4) [37,38,39,40]. Each community of Reactome pathways identified by the walktrap clustering was then aggregated into a new grouped pathway annotation. These grouped pathway annotations were then used for the network partitioning calculations.

### 2.14. Feature–Feature Networks and Partitioning

Each MW dataset that was used for outlier detection (711) was also checked against the list of datasets used in the prediction of pathways and enrichment by Huckvale et al. (resulting in 137 datasets) [34]. The various correlation measures were calculated between all features (see Correlation Methods). For any given correlation method, we generate the feature–feature network for that dataset–correlation method combination. The dataset correlations were transformed to partial correlations. From the distribution of partial correlation values, we consider the fraction of values that make up the 2.5% of the tail values (for a total of 5%) as the significant partial correlations that can be used as actual edges in the network. The network is then trimmed to only the edges that have a positive weight.

For each feature annotation (see Feature Annotations), we calculate three sums of the edge weights.

1. The total sum of edge weights for all edges with features that are annotated to one or more of the annotations (*annotated*).
2. The *within* annotation edge weight sum, where both start and end nodes are annotated to the same annotation.
3. The *outer* annotation edge weight sum, where the start node is part of the annotated set, and the end node is annotated to one of the other annotations.

The partitioning ratio (or q-ratio) is calculated as follows:

$$Q=\sum_{i=1}^{annot}\frac{within_{i}}{annotated}-{\left(\frac{outer_{i}}{annotated}\right)}^{2}$$

The partitioning ratio was originally designed as a method to determine the optimal clustering of networks, where each member of the network has only a single label [41,42]. In those cases, the partitioning ratio should range between 0 and 1 for non-partitioned and fully partitioned networks, respectively. The grouped Reactome pathways still have shared metabolite features, and therefore, the partitioning ratios have a much wider range of values. However, we expect that better partitioning of the network will be reflected by more-positive partitioning ratios.

Partitioning ratios were compared across correlation methods using a paired *t*-test, where methods were paired by the dataset. *p*-values were adjusted using the Bonferroni correction.

### 2.15. Changes in Correlation Due to Changes in Dynamic Range and Imputation

We created a simulated dataset as noted in Table 1 (lod, noise-3), starting with a sample from a random log-normal distribution (lod). Uniform noise was added via random normal distribution (noise-3) to create 100 samples from the base distribution, values were transformed to normal space, and then $log_{10}$ was applied to have a representation of orders of magnitude and dynamic range. For any maximum level of censoring to be applied, a uniform distribution sample is generated on the range of 0–max with 100 values. Data censoring was applied by taking the minimum observed value for a sample and adding the censoring value from the uniform distribution. Any values in the sample below the censoring value are set to missing (NA).

Correlations were calculated between samples when no missing values were present (*reference*), and then again after censoring (*trimmed*). Two different correlation methods were used: ICI-Kt and Pearson correlation. Imputation for Pearson correlation involved replacing all missing values with ½ the lowest observed value in the dataset after censoring. Differences between the reference and trimmed correlations were calculated, as well as the difference in the absolute value of differences in ICI-Kt and Pearson imputed.

### 2.16. Performance and Efficiency Evaluations

We compared the performance of our Rcpp mergesort implementation to the base R cor function using both “pearson” and “kendall” methods on a single core with increasing numbers of features, ranging from 100 to 5000, in increments of 100.

To verify the parallel implementation in R, we created a fake dataset with 10,000 features and 400 samples via rnorm. Using Rscript to run each iteration in a clean R session, we ran calculated ICI-Kt correlations between the 400 samples for this dataset with an increasing number of cores, ranging from 1 to 12, timing the calculation for each one, and logging the memory used from the ICIKendallTau::log_memory. The results are returned as a data.frame to save memory and time generating the matrix. For Python, we created a similar dataset using numpy.random.randn, and then ran it on all cores, as the current package does not allow setting the number of cores in the multiprocessing.Pool creation. We logged Python memory usage using a bash script writing memory use to a file every 5 s. In both cases, the memory usage prior to starting calculations was recorded so that the increased memory usage specific to calculating the ICI-Kt results and saving them could be noted.

### 2.17. Data Processing

All data processing and statistical analysis used R v 4.4.1 [25], Bioconductor v 3.20 [43], and Python v 3.13. JSONized MW files were read in using jsonlite v 2.0.0 [44]. Metabolite feature id and sample id cleaning used janitor v 2.2.1 [45] and dplyr v 1.1.4 [46]. Plots were generated using, either singly or in combination, the packages: ggplot2 v 4.0.0 [47]; ggforce v 0.5.0 [48]; and patchwork v 1.3.2 [49]. Analysis workflows were coordinated using targets v 1.11.4 [50]. Reactome pathway manipulation and aggregation used categoryCompare2 v 0.200.4 [35]. Outlier detection used visualizationQualityControl v 0.5.6 [31].

## 3. Results

### 3.1. Datasets

We are aware of only one previous investigation of the causes of missingness in metabolomics datasets [41]. In Do et al. [41], the authors showed that there was a limit-of-detection (LOD) effect, with a dependence on the day the samples were run. Unfortunately, the KORA4 metabolomics dataset from Do et al. is not publicly available, so we could not attempt to redo their analysis of missing values with the same dataset.

Given the number of projects and analyses available in the Metabolomics Workbench (MW), we sought to obtain a large number of individual datasets (MW analyses) from MW to evaluate both the phenomenon of left-censorship and the information-content-informed Kendall-tau methodology.

**Figure 2:**
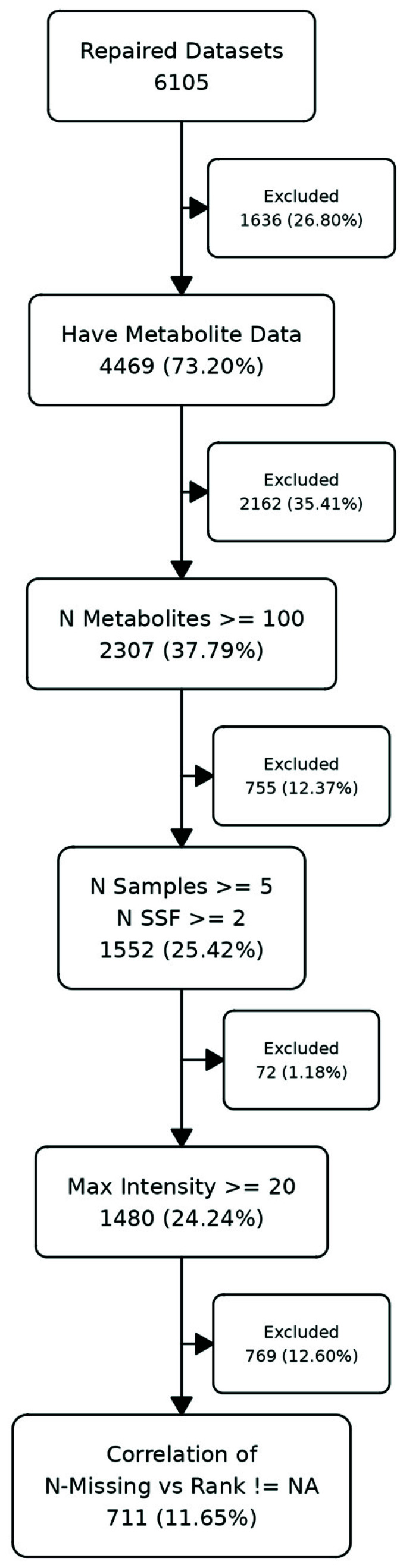
Number of datasets remaining after each level of filtering and/or checking.

In Figure 2, we provide a summary of the number of datasets remaining after filtering for various attributes (see Methods). From the starting 6501, we were able to retain 711 for this study. There were 1636 datasets that had no metabolite abundance data, or the metabolite data was not easily parseable or downloadable from external text files. A further 2162 datasets were excluded due to having <100 metabolites. In total, 755 were removed because they had <5 samples in at least one group of subject sample factors (SSF) or <2 SSF. A total of 72 remaining datasets had a maximum intensity < 20, indicating either being log-transformed or having a very small dynamic range. Finally, 769 datasets were removed due to either having no missing values, or the calculation of a correlation of rank with the number of missing values across samples returning an NA value.

### 3.2. Left-Censoring as a Cause for Missingness

**Figure 3:**
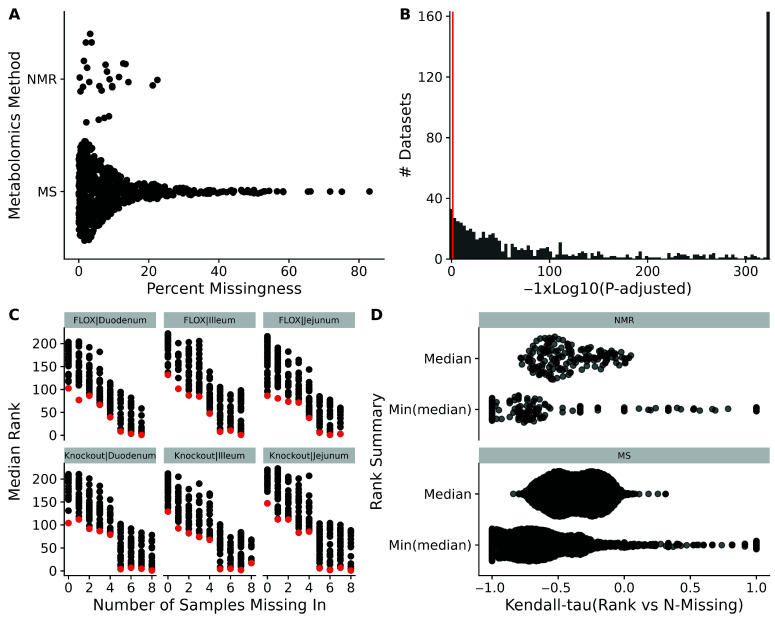
Missingness in datasets. (**A**) Sina plot of the percent missing values in NMR and MS datasets. (**B**) Histogram of log10 adjusted *p*-values versus number of datasets from the binomial test testing if non-missing values are more likely to be below the median rank when the metabolite has missingness in the dataset. The red line indicates an adjusted *p*-value of 0.05. Adjusted *p*-values of 0 were replaced with the lowest observed non-zero adjusted *p*-value. (**C**) Median (black) and minimum median (red) rank of metabolite abundances across factor groups of samples vs. the number of samples for which the metabolite had a missing value for dataset AN001074. (**D**) Sina plots of the Kendall-tau correlation of the median rank and minimum median rank with the number of missing samples across all datasets.

One would wonder just how many missing values are present in metabolomics datasets, and if their missingness is primarily due to left-censorship or some other phenomenon. For the 711 datasets examined in this work, the percentage of missingness ranged from near-zero (we required at least one missing value to keep a dataset for further analysis) to 25% for nuclear magnetic resonance (NMR), and the majority of mass-spectrometry (MS) datasets had missingness in the 0–25% range, with some datasets having missingness > 80% (Figure 3A). Using a binomial test to check if missing values are more likely to have non-missing values ranked at ≤0.5 (i.e., below the median) of the non-missing values in that sample, we see that the vast majority (681 of 711) have an adjusted *p*-value ≤ 0.05, with over 160 having an extreme adjusted *p*-value (Figure 3B). We also checked if there is any relationship between the percentage of missing values and the binomial test *p*-values, but found none (Figure S1). We have included the analysis dataset measurement and chromatography information extracted from the MW file, the number of missing values, and the results of the binomial test for left-censorship for all MW datasets investigated in a Supplemental Excel File for those who may want to investigate the phenomenon for subsets of the datasets.

For each set of subject sample factors (SSFs) of a dataset, we calculated the median rank and number of measurement values missing across samples (i.e., N-Missing) for each metabolite. As shown in Figure 3C, there is a monotonically decreasing relationship between the median rank and the number of missing values for that metabolite. Moreover, as N-Missing decreases, there is clearly a minimum median rank below which the values do not cross, as illustrated by the red points (Figure 3C). As shown in Figure 3D, median rank and N-missing are negatively correlated across the majority of experiments, although there are more-positive correlations when using the minimum median rank. Given the results of the binomial test of missing ranks and this relationship of the minimum median value observed and N-Present, we believe that the majority of missing values in many metabolomics datasets are due to left-censorship.

This makes the ICI-Kt methodology appropriate for use in many metabolomics datasets containing missing values through the incorporation of the missing values below the LOD as useful information in the correlation calculation.

### 3.3. Comparison to Other Correlation Measures

We compared the ICI-Kt correlation to both Pearson and regular Kendall-tau-b correlations as calculated by the built-in R functions using simulated datasets with missing values (Figures S2–S4).

We created two samples with 1000 observations each, drawn from a log-normal distribution, added further variance using a uniform distribution, and sorted in each case to create a pair of X and Y samples with a correlation of 1 and −1 for both Pearson and Kendall-tau correlation measures. The true correlation for each of the Kendall and Pearson correlations was calculated, and then missingness was introduced in the lower range of values, up to half of the values (see Simulated Datasets).

**Figure 4:**
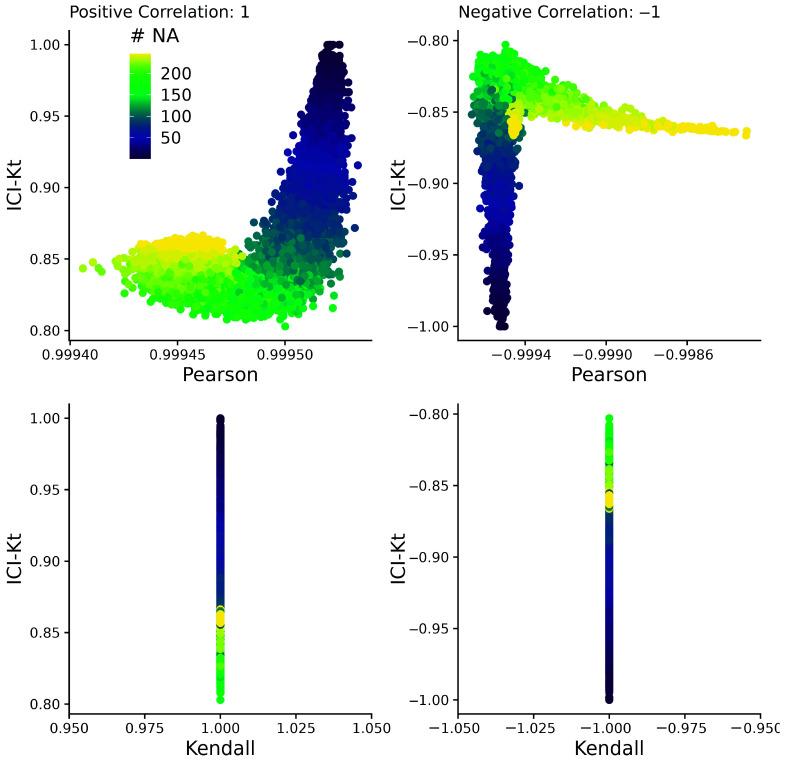
Comparing the correlation values obtained by Pearson, Kendall, and ICI-Kt correlation with an increasing number of missing values (# NA) from 0 to 500 in the bottom half of either sample for both positively (correlation = 1) and negatively (correlation = −1) correlated samples. Points are colored by how many points were set to missing on average between the two samples. A subset of 10,000 points was used for visualization.

In Figure 4 and Figure S5, we can see that as missing values are added, only the ICI-Kt correlation values change in any significant way, as illustrated by the wider range of ICI-Kt values on the y-axes versus the much narrower range of Pearson and Kendall tau correlation values on the x-axes. As the number of missing values increases, the ICI-Kt values drop or increase by up to 0.2. Similarly, Pearson correlation is also affected, but the degree of change in the correlation values is much less (notice the orders of magnitude differences in the *x*-axis scales compared to the *y*-axis), on the order of only 0.005 for both cases.

Adding outlier points at the high end of the distribution to one of the samples causes very odd discrete patterns to appear in the negative Pearson correlations (see Figures S6 and S7). Again, the scale of the differences is much smaller in the Pearson correlations versus ICI-Kt. The negative Kendall correlations are unaffected by the outliers, in large part due to being a rank-based correlation. Likewise, the negative ICI-Kt correlations appear unaffected; however, the scale of changes seen in the negative Pearson correlations, if present in the negative ICI-Kt correlations, might simply be obscured by the changes due to missingness that are orders of magnitude larger.

These results demonstrate that the ICI-Kt correlation has quantitative sensitivity to missing values over the normal Kendall-tau correlation and linear Pearson correlation, where points with missing values are ignored (pairwise complete).

### 3.4. Effect of Left-Censoring vs. Random Missing Data

**Figure 5:**
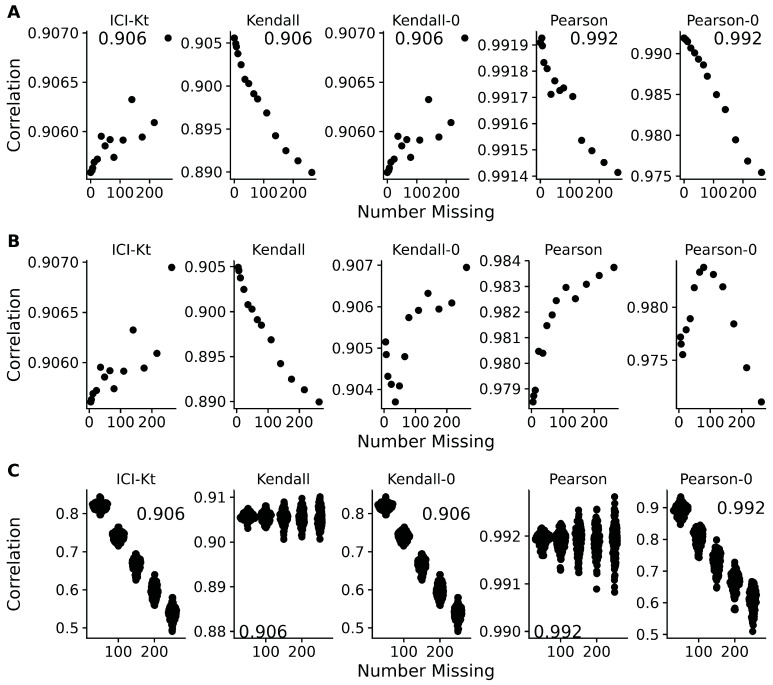
Effect of introducing missing values either from a cutoff (**A**,**B**) or randomly (**C**) on different measures of correlation, including ICI-Kt, Kendall with pairwise complete, Kendall replacing missing with 0, Pearson with pairwise complete, and Pearson replacing missing with 0. (**A**) Missing values introduced by setting an increasing cutoff. (**B**) Missing values introduced by setting an increasing cutoff, and then log-transforming the values before calculating correlation. (**C**) Missing values introduced at random. For the random case, each sample of random positions was repeated 100 times. Pay attention to the different *y*-axis ranges across graphs, with (**A**,**B**) graphs having much smaller *y*-axis ranges compared to (**C**).

Figure 5 demonstrates the effect of introducing left-censored versus random missingness in five different measures of correlation, including the ICI-Kt, the normal Kendall-tau with pairwise-complete entries, the normal Kendall-tau replacing missing with 0, Pearson with pairwise-complete entries, and Pearson replacing missing with 0. The ICI-Kt correlation demonstrates a slight increase from the starting 0.90 correlation value, with growing left-centered missingness caused by a slight reinforcement of the correlation, while with growing random missingness, the ICI-Kt correlation drops precipitously due to the large increase in discordant pairs caused by the random missing values. The normal Kendall tau correlation using only pairwise-complete entries has a small decrease in the correlation value with growing left-centered missingness caused by a loss of supporting pairs, while this correlation has a near-constant average value with growing random missingness. The normal Kendall tau correlation replacing missing with 0 has identical behavior to the ICI-Kt correlation. In contrast to ICI-Kt, the Pearson correlation calculated using only pairwise-complete entries is practically constant (i.e., range of 0.004 or less) over growing left-centered and random missingness. When replacing missing values with zero, Pearson correlation demonstrates a small decrease in the correlation value with growing left-centered missingness, due to the zero values causing some deviation from linearity. Pearson correlation drops precipitously with growing random missingness, with a magnitude similar to that of the ICI-Kt and normal Kendall tau replacing missing with 0. Overall, the ICI-Kt and the normal Kendall-tau replacing missing with zero have the desirable characteristics of maintaining the correlation with growing left-centered missingness, while sharply dropping the correlation with growing random missingness. In this special case where zero is lower than all of the values in the dataset, ICI-Kt and Kendall-tau replacing with zero result in identical correlation values, as shown in the bottom panels of Figure S8A,C. In a naive treatment of the left-centered missing data, if the values below the cutoff are set to missing, followed by log-transforming the values and subsequently setting missing values to 0, then the Kendall tau correlation replacing missing with 0 will show some very odd patterns at low intensity cutoffs due to the introduction of discordant pairs. Likewise, Pearson correlation replacing missing with 0 shows a parabolic effect with increasing missing values.

A common way missing data is handled in correlation calculations is to ignore them completely and use the pairwise-complete cases to calculate the Pearson correlation coefficient. As shown in Figure 5C, this results in a complete misestimation of the changed correlative structure introduced by random entries. ICI-Kt, in contrast, incorporates the missingness in a sensical way, and the resulting correlation values fall as random entries are introduced.

### 3.5. Differences in Dynamic Range and Correlation

Another way that missing values appear is due to changes in dynamic range between samples, as some samples have features with higher values, and the fixed dynamic range of the instrumentation results in features with lower values to be “missing” in those samples. We created a set of 100 simulated samples with uniform noise on the log-scale, with relatively constant dynamic ranges, and introduced changes to the overall dynamic range using a random censor at varying levels (see Implementation). Possible different levels of censoring based on dynamic range were checked by first determining how many missing values would be introduced in each sample as the dynamic range was increased in increments of 0.1 (see Figure S9). Based on the number of values being censored, limits of 0.5, 1, and 1.5 were selected, representing low, medium, and high variability of the dynamic range.

**Figure 6:**
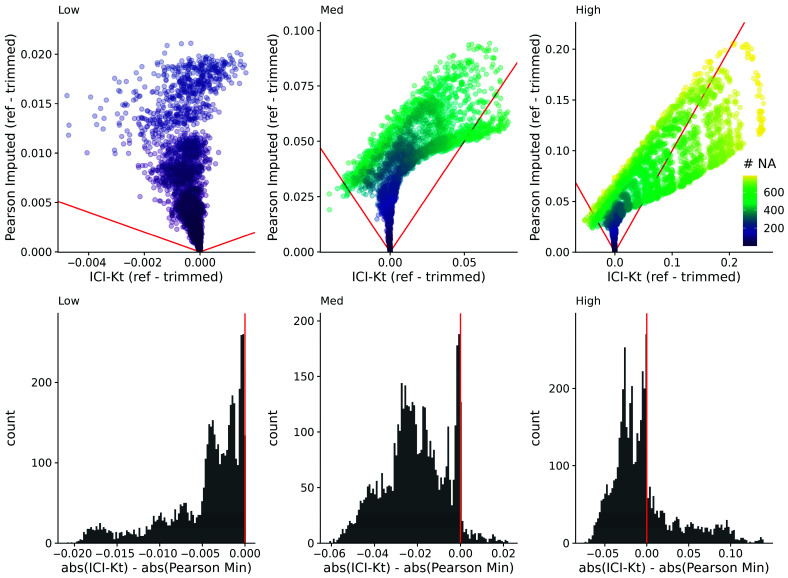
(**Top**) Difference of reference—trimmed ICI-Kt correlation vs. Pearson imputed using 1/2 the minimum value in the dataset. Low, med, and high indicate the level of variability in dynamic range, using 0.5, 1, and 1.5, respectively. Red lines indicate slope of −1 and 1. Color indicates the maximum number of missing values between the two samples being correlated. (**Bottom**) Differences in the absolute value of reference minustrimmed differences between ICI-Kt and Pearson imputed correlations.

For each level of possible missingness introduced by changes to the dynamic range, correlation across all samples was calculated using all values (reference), as well as after missingness was added (trimmed), and using Pearson correlation with global imputation (Pearson imputed), or ICI-Kt. Figure 6 demonstrates that it is only as the number of missing values in one of the samples approaches 50% or more (500 of 1000 features) that the Pearson correlation with global imputation gives correlation values closer to the known correlation with no missing values in any appreciable amount (points below the red lines in the top panels, and to the right of the red line in the histograms in the bottom panels). Points above the lines with slopes of −1 and 1 indicate that the difference of reference—trimmed is smaller in the ICI-Kt correlations, and points below the lines indicate the difference is larger in the ICI-Kt correlations. This is further emphasized by the majority of the values being to the left of the line at 0 in the difference histograms.

### 3.6. Utility for Metabolomics Datasets

Having established that many metabolomics datasets with missing values are present due to left-censorship (see Figure 3), we analyzed how the ICI-Kt methodology compares to other methods for outlier removal and for the generation of feature–feature networks.

For outlier removal evaluation, we directly used differential analysis of metabolites across conditions. For each correlation method, outlier samples within each SSF were determined and removed, and an F-test was conducted using the limma package across SSFs. The fraction of metabolites that were differential after outlier removal was determined, and a *t*-test was used to evaluate pairwise comparisons of methods to see if any differences were significant.

Table 2 shows the fractions of significant metabolites after removing outlier samples using each method. Table 3 shows the statistical results of the pairwise comparisons of each method based on the significant fractions. Both tables show that while there is a significant change in the fraction of significant metabolites after removal of outlier samples, the actual average differences are very small.

In feature–feature network generation, we evaluated the differences in partitioning ratios of metabolite features across aggregated Reactome pathways, after creating weighted feature–feature networks using the various correlation methods. Paired *t*-tests compared the methods, and are reported in Table 4 and Table S2, and graphed in Figure S10. Both the ICI-Kt complete and base variants show much larger positive differences in partitioning ratio compared to all other methods, including the base Kendall-tau. This implies that the gains in the partitioning ratio are not only due to using a rank-based correlation.

### 3.7. Computational Performance and Efficiency

Timing the calculation of correlation using Pearson, ICI-Kt, and Kendall-τ in R with increasing numbers of features, each shows the expected algorithmic complexity of O(n), O(nlog(n)), and O(n^2^), respectively (see Figure S11). Therefore, while not as fast as Pearson correlation, ICI-Kt does match the expected complexity in practice, using the currently fastest method known for the calculation of the Kendall-τ correlation.

When multiple compute cores are available, furrr (and the underlying future package) makes it rather trivial to split up the computation across cores and recombine them at the end. Figure S12 shows decreasing time taken for a dataset with 10,000 features and 400 samples (300 to 42 s), while increasing the overall memory used from 110 MiB to 11,790 MiB, as furrr does not have an easy way to have shared memory. There is less of a gain past five cores, likely due to the machine on which the calculations were run having six physically separate cores with two hyperthreaded cores each, one of which was always running the controlling process.

The Python version uses only 850 MiB of additional memory when using 12 cores, due to using the shared memory module for multiprocessing; however, the overall runtime is only slightly faster than the R version, at 42 s vs. 44 s, respectively.

## 4. Discussion

Left-censored distributions in analytical measurements of biological samples are common in biological and biomedical research because of the detection limits of the analytical instrumentation, which produces missing measurement values for all the analytes below these detection limits. As far as we are aware, previous efforts in this area are concerned with either 1: attempting to come up with better imputation methods prior to calculating correlation values; or 2: finding ways to still estimate the correlation in the face of missing values, generally by finding maximum-likelihood estimates. In the case of (1), there are many imputation methods, and new methods are still being developed, although they tend to be new combinations of old methods to handle the various types of missing values. For (2), the maximum-likelihood methods generally apply to Pearson and similar types of correlation, as they benefit from the use of regression in the presence of missing data. Alvo and Cabilio’s work from 1995 [51] is one of the only works we are aware of that attempts to create a general framework for rank-based statistics in the presence of missing data. But, in our understanding, their framework applies to data that is missing at random versus non-random missing values, as is the case for analytes that are below the detection limit. Additionally, there does not appear to be a software implementation of Alvo and Cabilio’s method available.

Although the actual implementation of the base ICI-Kt correlation metric involves a global imputation of missing values, our equations demonstrate a left-censorship interpretation of missing values as useful information within the calculated correlation. Furthermore, the addition of “local”, “global” and $\tau_{max}$ normalizations of the ICI-Kt correlation in combination with completeness provides additional interpretations of information content. The package functions ici_kt and ici_kendalltau, the default for the calculation of correlations between two samples and multiple samples, respectively, default to the “local” and “global” methods of handling ties, as laid out in Section 2.3. Finally, the availability of the binomial left-censorship test ensures the application of the ICI-Kt methodology when it is appropriate. Future directions for this methodology are to explore handling several additional statistical conditions. Specifically, the methodology and the codebases will be expanded to handle right-censorship, which we expect to be straightforward. In addition, approaches will be explored to handle data with combinations of right-censorship, left-censorship, and missing at random. We expect the expansion of the methodology to handle a combination of both right- and left-censorship, also known as double-censorship, to be tractable. However, combinations of left-, right-, or double-censorship with missing at random may not have good solutions by this methodological approach.

Global imputation methods rely on the assumption that samples have similar dynamic ranges of detection, and thus, that an imputed value should be comparable between samples. However, the dynamic range of detection is often variable across samples. For complex analytical techniques often used in omics experiments, the variability in the dynamic range of detection can be quite high. Under these circumstances, the ICI-Kt method provides more robust results, as compared to Pearson correlation with global imputation. This holds true for low, medium, and high variability in dynamic range across samples. While technically the information content is not changed, the quality of the interpretation of the information is better within the ICI-Kt correlation metric, as shown by the explicit equations in Section 2.1 and Section 2.2 in comparison to Pearson correlation with global imputation.

In the case of using sample–sample correlation to detect outliers, imputation does not solve any of the issues related to discovering outliers, as it should be applied after outlier samples are removed; otherwise, the imputed values may not be useful. When used to create feature–feature networks based on partial correlations derived from the feature–feature correlations, ICI-Kt-based methods showed the best partitioning of features based on predicted pathway annotations. As far as we know, information-content-informed Kendall-tau (ICI-Kt) is the first correlation method that explicitly attempts to utilize non-random missing values that occur due to being below the detection limit. ICI-Kt explicitly treats left-censored missing values as correlative information while preserving the full deleterious effects of missing at random values on the correlation metric. Moreover, ICI-Kt can be combined with measurement completeness to create a composite metric that is quite sensitive to overall data quality on a sample-specific level. Also, this ICI-Kt × completeness metric may have applications in cluster detection of single-cell omics datasets.

ICI-Kt has been directly used in the analysis of metabolomics and other omics datasets, providing useful biological and biomedical insight. In Mitchell et al. 2021, a sterol feature-limited sample–sample ICI-Kt correlation matrix (Figure 5A in the paper) illustrates at least two distinct groups of resected human non-small lung cancer (NSCLC) samples not specific to histological subtypes [5]. Also, a feature–feature ICI-Kt correlation matrix (Figure 4 in the paper) illustrates distinct patterns of high correlation between specific groups of phosphosphingolipids and glycerophospholipids [5]. In Braun et al. 2025, a sample–sample ICI-Kt correlation matrix (Figure 2A in Braun et al. paper) clearly separates cerebrospinal-fluid aneurysmal subarachnoid hemorrhage, plasma aneurysmal subarachnoid hemorrhage, and control plasma cytokine samples, which is not illustrated in the principal component analysis [52]. Statistically significant cytokine correlations between cerebrospinal fluid and plasma samples (illustrated in Figure 3 of the paper) identified CXCL12, IL-15, and SAA1 as potential biomarkers for neurysmal subarachnoid hemorrhage and potential therapeutic targets [52]. In Anspach et al. 2026, feature–feature ICI-Kt correlation matrix analyses enable multi-omics integration of transcriptomics, metabolomics, and lipidomics datasets derived from resected tumor and nearby non-tumor paired samples from human patients with steatohepatitic hepatocellular carcinoma [53]. Over 300 novel gene–metabolite and gene–lipid correlative relationships were identified in these ICI-Kt-driven analyses [53].

The implementations of the ICI-Kt method in the available R and Python packages provide a rich set of options for customizing the correlation calculation for a variety of use cases and interpretations. These packages handle missing values in log-transformed data in a safe manner and have O(nlogn) performance, making them computationally practical for real-world omics datasets. Also, these packages provide multiprocessing implementations that take full advantage of modern multi-core central processing units.

As demonstrated with the datasets analyzed here, the “best” correlation-related metric will likely depend on the specific dataset and the specific data analysis step. Many factors affect this, especially correlation linearity and the modality of measurement value distributions. We would humbly suggest that for most metabolomics datasets, the application of several correlation-related metrics simultaneously would be the best approach for outlier detection in quality control and quality assessment steps. Where one metric lacks outlier detection sensitivity, another metric will prove sensitive. Therefore, ICI-Kt and associated composite metrics should be considered as useful additions to the omics data analysis toolkit.

## 5. Conclusions

Missing values due to left-censorship are common in metabolomics datasets, as demonstrated across 711 publicly deposited datasets in the Metabolomics Workbench. To better address the effects of left-censored missing values, the ICI-Kt methodology directly interprets missing values within a Kendall-tau rank-based correlation metric. These new ICI-Kt metrics demonstrate superior performance to Pearson correlation-based metrics with respect to capturing meaningful biological pathway relationships between metabolite features. ICI-Kt methods are implemented in easy-to-install open-source R and Python packages that demonstrate optimal computational performance while utilizing available cores on modern central processing units, making them practical for the analysis of high-feature and high-sample omics datasets. Thus, ICI-Kt methods are available for general use in omics data analysis, especially metabolomics data analysis.

## Figures and Tables

**Table 1:** Parameters used for various simulated data.

Dataset	Distribution	N	Mean	SD	Range
perfect	log-normal	1000	1.0	0.5	
noise-1	uniform	1000			−0.5–0.5
outlier	log-normal	5	1.2	0.1	
realistic	log-normal	1000	1.0	0.5	
noise-2	normal	1000	0.0	0.2	
lod	log-normal	1000	1.0	0.5	
noise-3	normal	1000	0.0	0.2	

**Table 2:** Summary statistics of the fraction of significantly different features after removing outliers detected using each correlation method.

Method	Mean	SD	Median	MAD
icikt	0.457	0.338	0.454	0.482
icikt_complete	0.457	0.336	0.450	0.480
pearson_log1p	0.455	0.337	0.450	0.487
kt_base	0.455	0.337	0.460	0.478
pearson_log	0.453	0.337	0.450	0.475
pearson_base	0.450	0.338	0.442	0.483
pearson_base_nozero	0.448	0.338	0.441	0.480
original	0.443	0.338	0.436	0.484

**Table 3:** Paired *t*-test statistical results comparing the fraction of significant metabolite features after removing outlier samples using different methods. Adjusted *p*-values were calculated using the Bonferroni method.

Comparison	Difference	*p*-Value	*p*-Adjusted
icikt v original	0.0137	7.9 × 10^−13^	3.5 × 10^−11^
icikt_complete v original	0.013	1.5 × 10^−11^	6.9 × 10^−10^
kt_base v original	0.0104	1.1 × 10^−9^	4.9 × 10^−8^
pearson_log1p v original	0.011	2.7 × 10^−9^	1.2 × 10^−7^
pearson_log v original	0.00964	4.1 × 10^−9^	1.8 × 10^−7^
icikt v pearson_base	0.00819	3.8 × 10^−8^	1.7 × 10^−6^
icikt v pearson_base_nozero	0.00949	5.5 × 10^−8^	2.5 × 10^−6^
icikt_complete v pearson_base_nozero	0.00888	1.5 × 10^−6^	6.8 × 10^−5^
icikt_complete v pearson_base	0.00758	3.6 × 10^−6^	1.6 × 10^−4^
pearson_base_nozero v kt_base	−0.00625	2.7 × 10^−5^	1.2 × 10^−3^
pearson_base_nozero v pearson_log1p	−0.00687	1.0 × 10^−4^	4.7 × 10^−3^
pearson_base_nozero v pearson_log	−0.00547	2.0 × 10^−4^	9.0 × 10^−3^
pearson_base v kt_base	−0.00495	2.3 × 10^−4^	1.0 × 10^−2^
pearson_base v original	0.00547	2.6 × 10^−4^	1.2 × 10^−2^
pearson_base v pearson_log1p	−0.00558	2.8 × 10^−4^	1.3 × 10^−2^

**Table 4:** Paired *t*-test statistical results comparing the partitioning ratio of networks generated by the various correlation methods. Adjusted *p*-values were calculated using the Bonferroni method.

**Comparison**	**Difference**	***p*** **-Value**	***p*** **-Adjusted**
icikt_complete v pearson_log	2.3	2.7 × 10^−12^	5.7 × 10^−11^
icikt v pearson_log	2.35	3.9 × 10^−12^	8.2 × 10^−11^
icikt v pearson_base_nozero	2.51	4.1 × 10^−12^	8.6 × 10^−11^
icikt_complete v pearson_base_nozero	2.45	7.5 × 10^−12^	1.6 × 10^−10^
icikt v kt_base	1.7	1.2 × 10^−10^	2.6 × 10^−9^
icikt_complete v kt_base	1.63	3.7 × 10^−10^	7.7 × 10^−9^
icikt v pearson_log1p	1.15	8.6 × 10^−9^	1.8 × 10^−7^
icikt_complete v pearson_log1p	1.08	9.2 × 10^−9^	1.9 × 10^−7^
icikt v pearson_base	1.05	6.6 × 10^−8^	1.4 × 10^−6^
icikt_complete v pearson_base	0.976	6.8 × 10^−8^	1.4 × 10^−6^
pearson_base v pearson_base_nozero	1.41	7.7 × 10^−7^	1.6 × 10^−5^
pearson_base_nozero v pearson_log1p	−1.33	1.7 × 10^−6^	3.7 × 10^−5^
pearson_base v pearson_log	1.31	3.1 × 10^−5^	6.6 × 10^−4^
pearson_log1p v pearson_log	1.18	3.4 × 10^−4^	7.1 × 10^−3^
pearson_base v kt_base	0.62	6.5 × 10^−4^	1.4 × 10^−2^

## Data Availability

The code and data used in the results for this manuscript are available on Zenodo at the following DOI: https://doi.org/10.5281/zenodo.18625643 (accessed on 17 February 2026). GitHub repository for the R ICIKendallTau package: https://github.com/moseleyBioinformaticsLab/ICIKendallTau (accessed on 17 February 2026). GitHub repository for the Python icikt package: https://github.com/moseleyBioinformaticsLab/icikt (accessed on 17 February 2026). Python Package Index: https://pypi.org/project/icikt/ (accessed on 17 February 2026).

## Supplementary Materials

The following supporting information can be downloaded at https://www.mdpi.com/article/10.3390/metabo16040245/s1: Figure S1. Percent missing values in each dataset compared to the *p*-values from the left censored binomial test.; Figure S2. Comparison of ICI-Kt and Pearson correlations for perfectly positive and negatively correlated samples, systematically replacing values with NA; Figure S3. ICI-Kendall-tau correlation as missing values are varied between two samples; Figure S4. Kendall-tau correlation as missing values are varied between two samples and replaced with 0 before calculating Kendall-tau.; Figure S5. Difference of estimated correlation with missingness introduced compared to a reference correlation of 1 for the positive or -1 for the negative case, as a function of the average number of missing entries in X and Y sample (# NA); Figure S6. More variance was introduced at one end of the log-normal distribution in one sample to create a small percentage of outlier points compared to the other samples; Figure S7. More variance was introduced at one end of the log-normal distribution in one sample to create a small percentage of outlier points compared to the other samples; Figure S8. Effect of introducing missing values from a cutoff (A & B) or randomly (C) on different measures of correlation, including ICI-Kt, Kendall with pairwise complete, Kendall replacing missing with 0, Pearson with pairwise complete, and Pearson replacing missing with 0; Figure S9. Number of missing values in each of the 100 samples as a function of changing the dynamic range of the values in the sample by increasing the lower limit of detection; Figure S10. Boxplot and sina plots of paired differences of partitioning ratios across datasets; Figure S11. Time in seconds needed as a function of the number of features, with a fitted line for the assumed complexity for each of the methods tested, including R’s Pearson correlation, the ICI-Kt mergesort, and R’s Kendall-tau correlation algorithm; Figure S12. (A): Time in seconds as the number of compute cores is increased. (B): Additional memory used as the number of compute cores is increased; Table S1. The statistical results of the pairwise comparisons of each method based on the significant fractions after removing outliers; Table S2. Paired *t*-test statistics comparing all the methods to each other.

## Abbreviations

The following abbreviations are used in this manuscript:

ICI-Kt	Information-content-informed Kendall-tau
EDA	Exploratory data analysis
PCA	Principal component analysis
MW	The Metabolomics Workbench
SSF	Subject sample factor
LOD	Limit of detection
NMR	Nuclear magnetic resonance
MS	Mass spectrometry
