# Supplementary material for: Information-Content-Informed Kendall-Tau Correlation Methodology: Interpreting Missing Values in Metabolomics as Potentially Useful Information
Source: Metabolites. 2026 Apr 4;16(4):245. doi: 10.3390/metabo16040245 (PMC13118274; doi:10.3390/metabo16040245)
Supplement: Supplementary file 1 [file metabolites-16-00245-s001.zip › supplemental_materials_2026-03-18_hnbm.pdf]

Supplemental Materials

Robert M Flight, Praneeth S Bhatt, and Hunter NB Moseley

2026-03-16 15:02:50.191047

Left Censoring as a Cause for Missingness

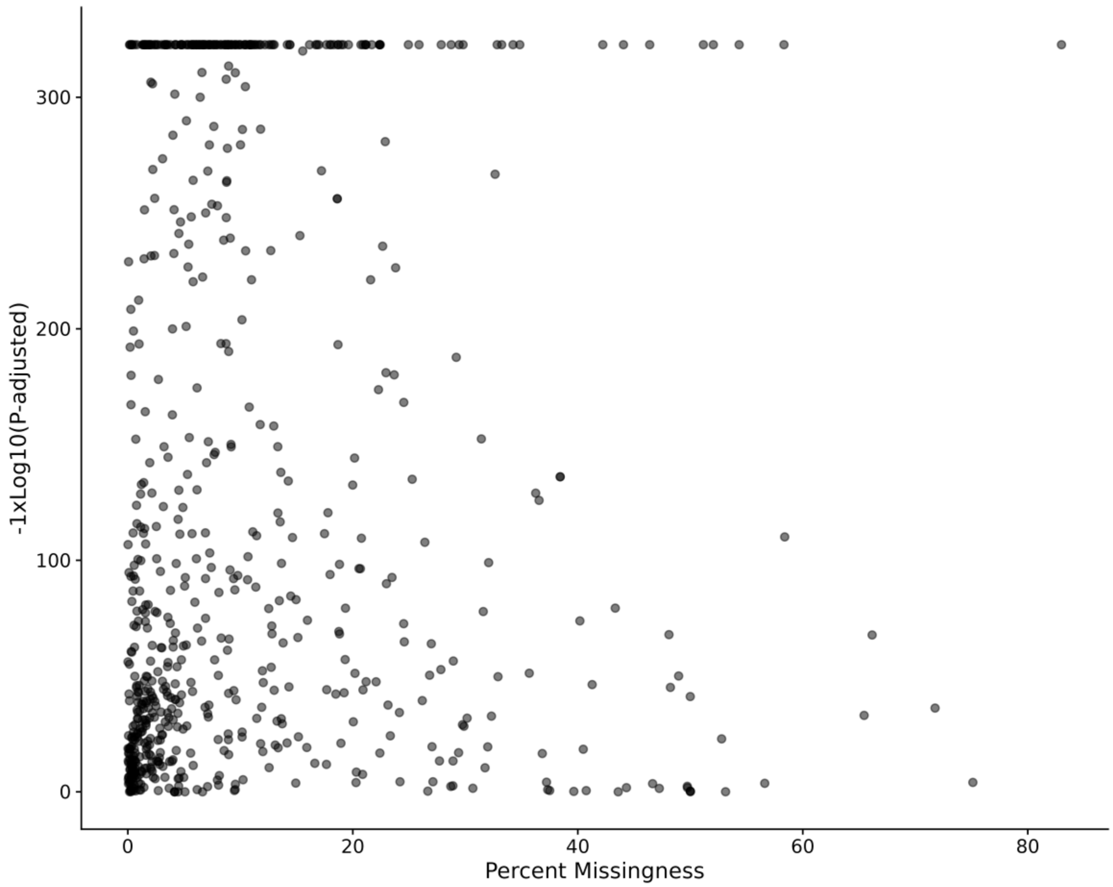

Figure S1. Percent missing values in each dataset compared to the p-values from the left censored binomial test.

Simulated Data

Simple Data Set

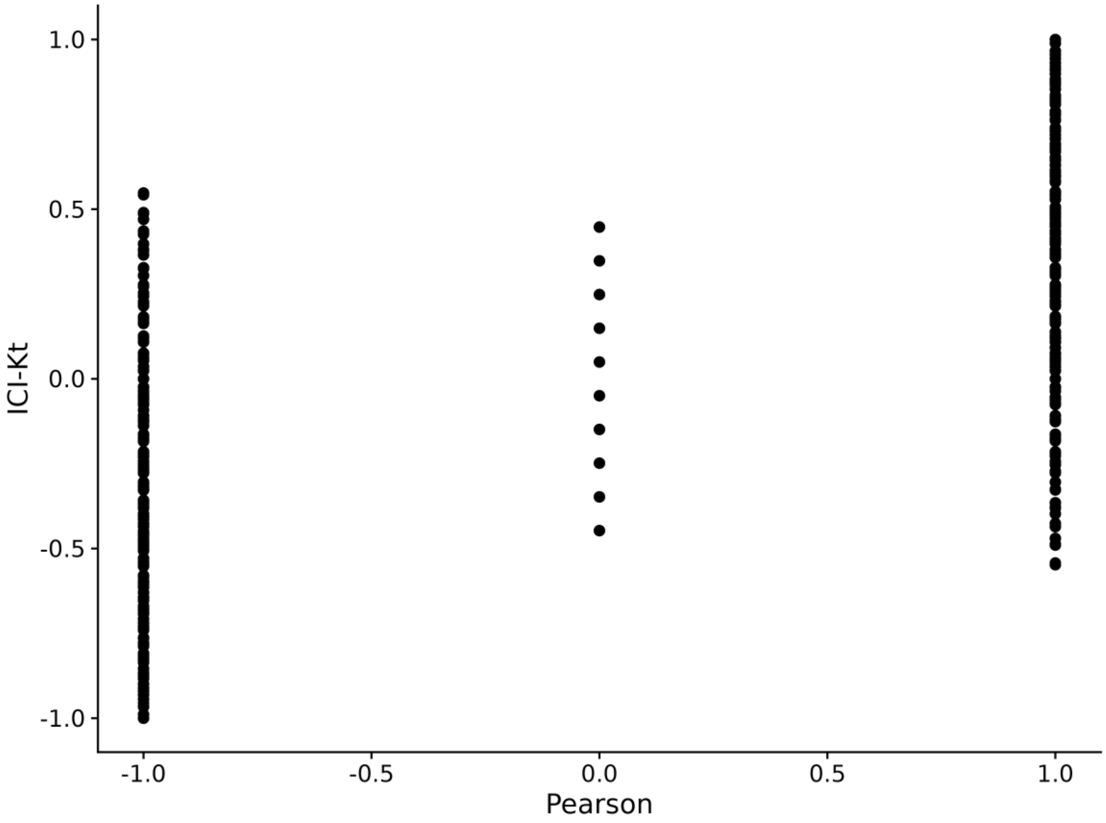

Figure S2. Comparison of ICI-Kt and Pearson correlations for perfectly positive and negatively correlated samples, systematically replacing values with NA. NA values from Pearson were replaced with zero for this comparison.

We can also examine the full set of positive and negative correlations generated as we vary the number of missing entries between two positively correlated samples and two negatively correlated samples. These distributions are shown in Figure S4. We can see that the distributions from both ICI-Kt and Kendall-tau are the same, which is expected given we replaced missing values (NA) with zero *within* the ICI-Kt code, and replaced missing values (NA) with zero prior to calculating Kendall-tau correlations.

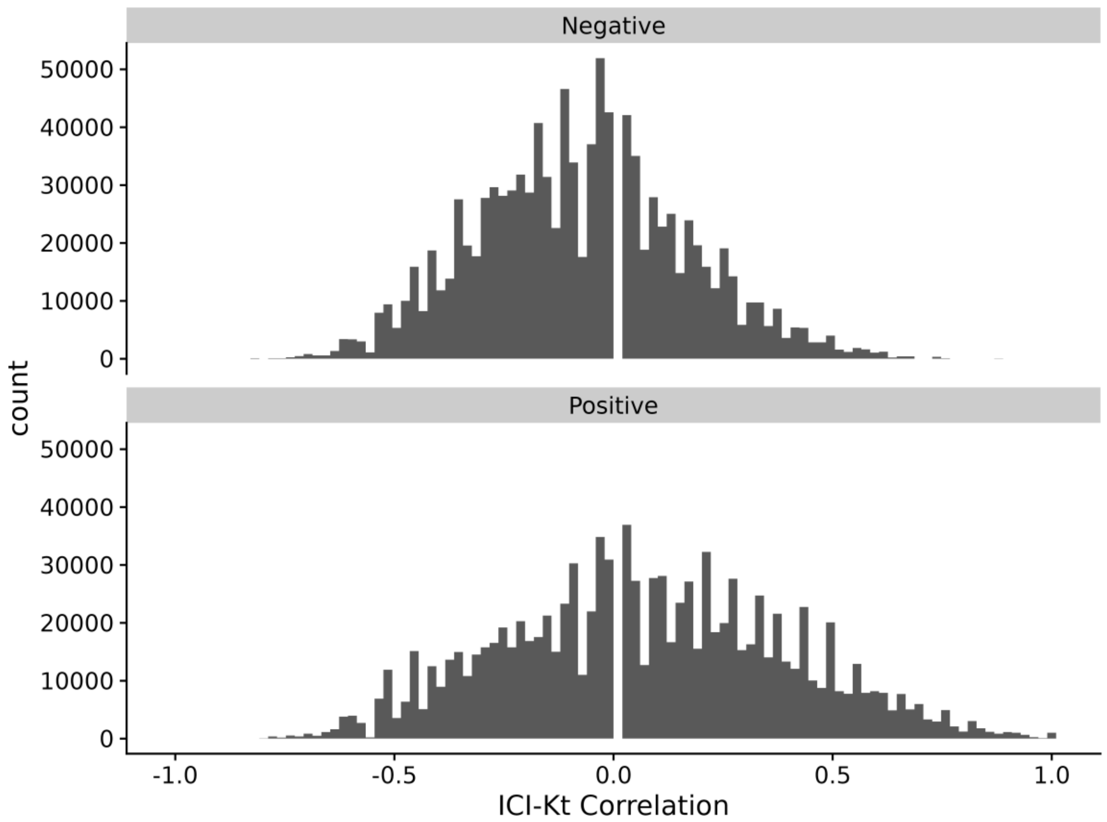

Figure S3. ICI-Kendall-tau correlation as missing values are varied between two samples.

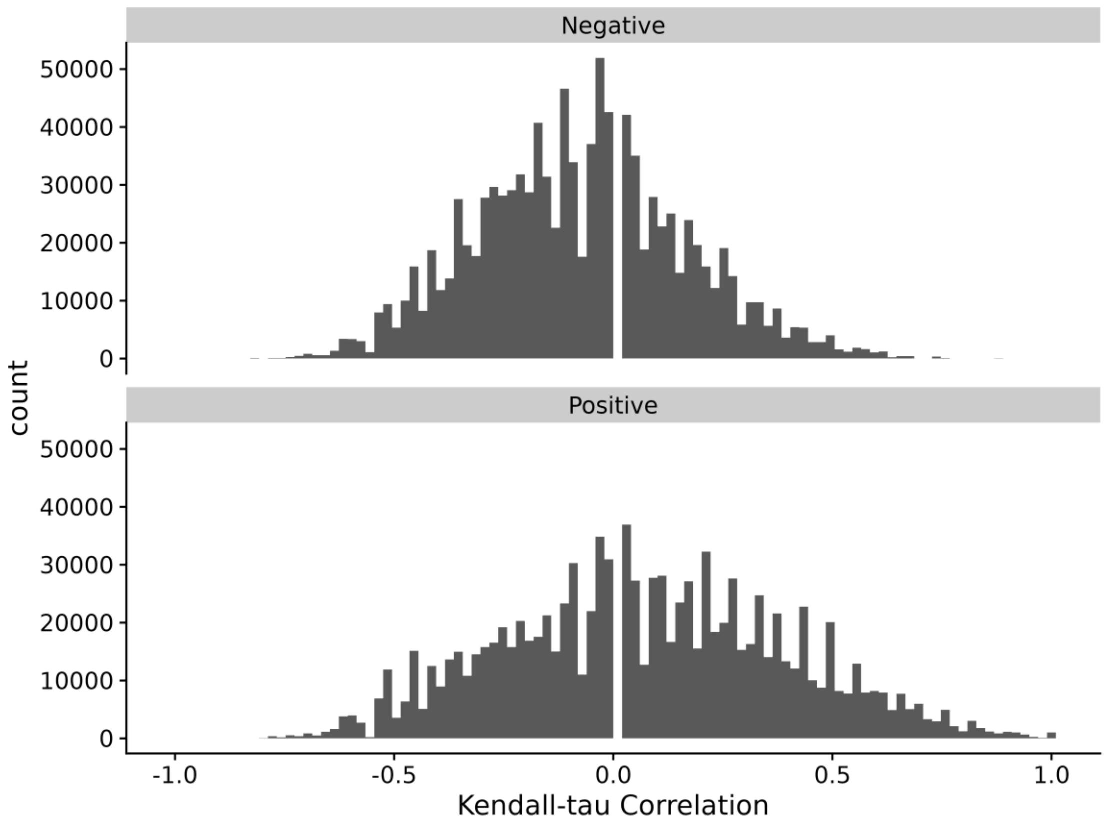

Figure S4. Kendall-tau correlation as missing values are varied between two samples and replaced with 0 before calculating Kendall-tau.

Comparison to Other Correlation Measures

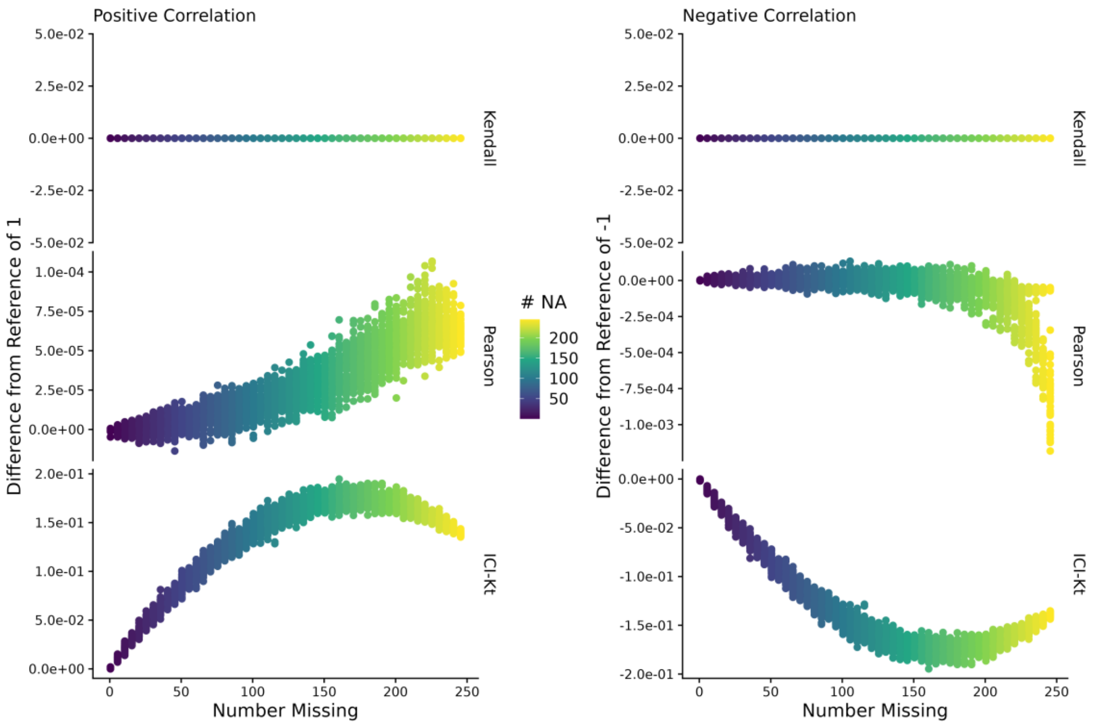

Figure S5. Difference of estimated correlation with missingness introduced compared to a reference correlation of 1 for the positive or -1 for the negative case, as a function of the average number of missing entries in X and Y sample (# NA). Points are colored by how many points are missing on average between the two samples X and Y.

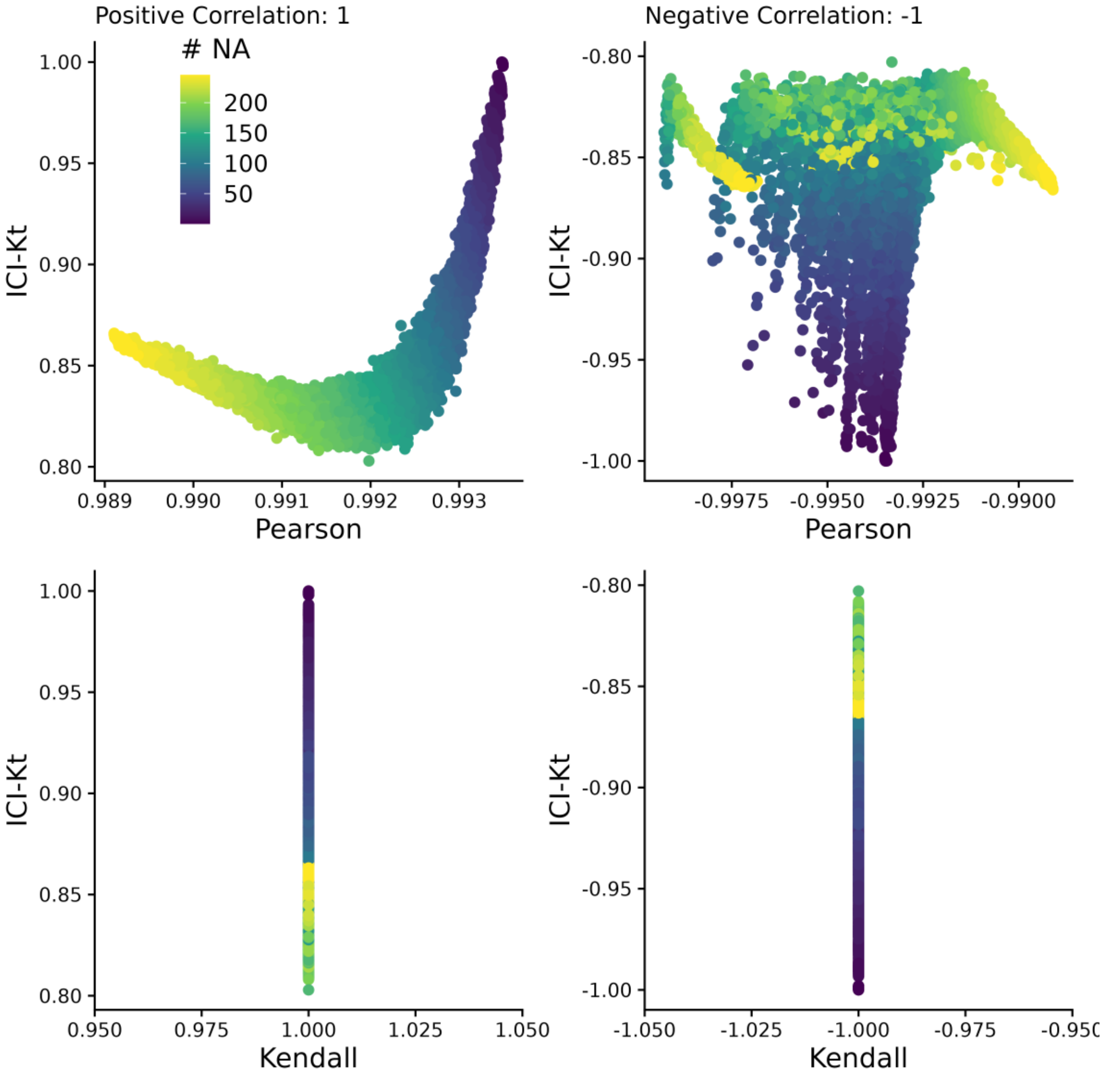

Figure S6. More variance was introduced at one end of the log-normal distribution in one sample to create a small percentage of outlier points compared to the other samples. This figure is comparing the correlation values obtained by Pearson, Kendall, and ICI-Kt correlation as an increasing number of missing values (0 - 500) in the bottom half of either sample for both positively (correlation = 1) and negatively (correlation = -1) correlated samples. Points are colored by how many points were set to missing on average between the two samples. A subset of 10,000 points was used for visualization.

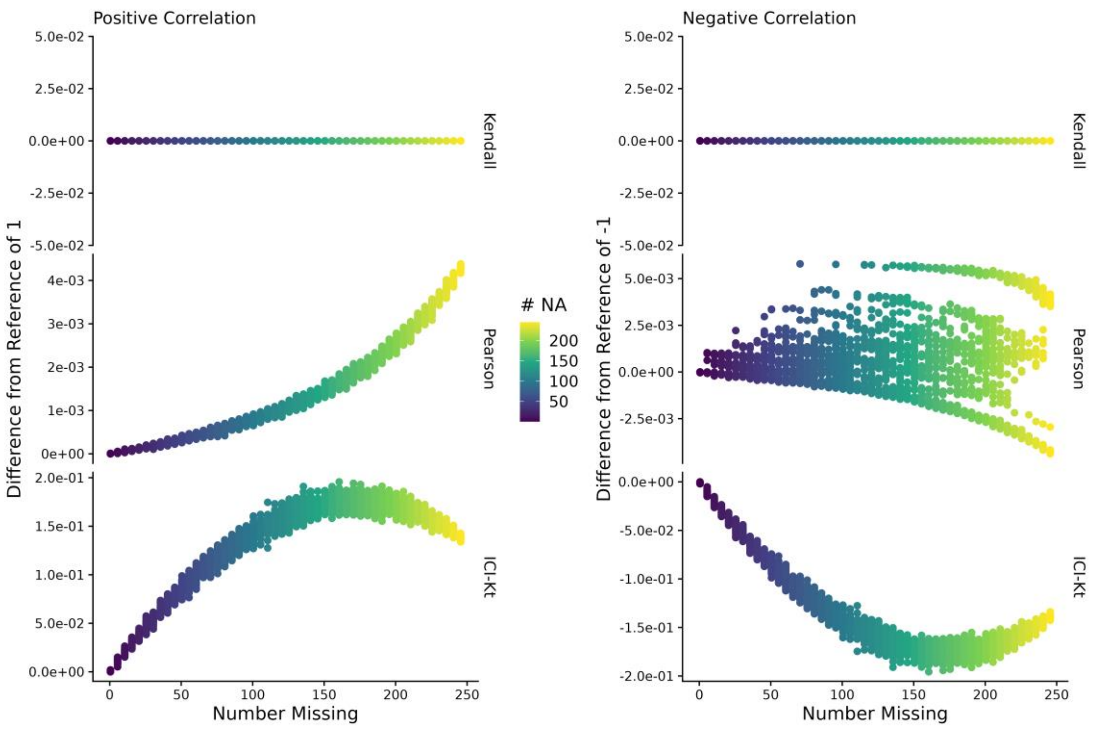

Figure S7. More variance was introduced at one end of the log-normal distribution in one sample to create a small percentage of outlier points compared to the other samples. This figure is showing the difference of estimated correlation with missingness introduced compared to a reference correlation of 1 for the positive or -1 for the negative case, as a function of the average number of missing entries in X and Y sample (# NA). Points are colored by how many points are missing on average between the two samples X and Y.

Semi-Realistic Data Set

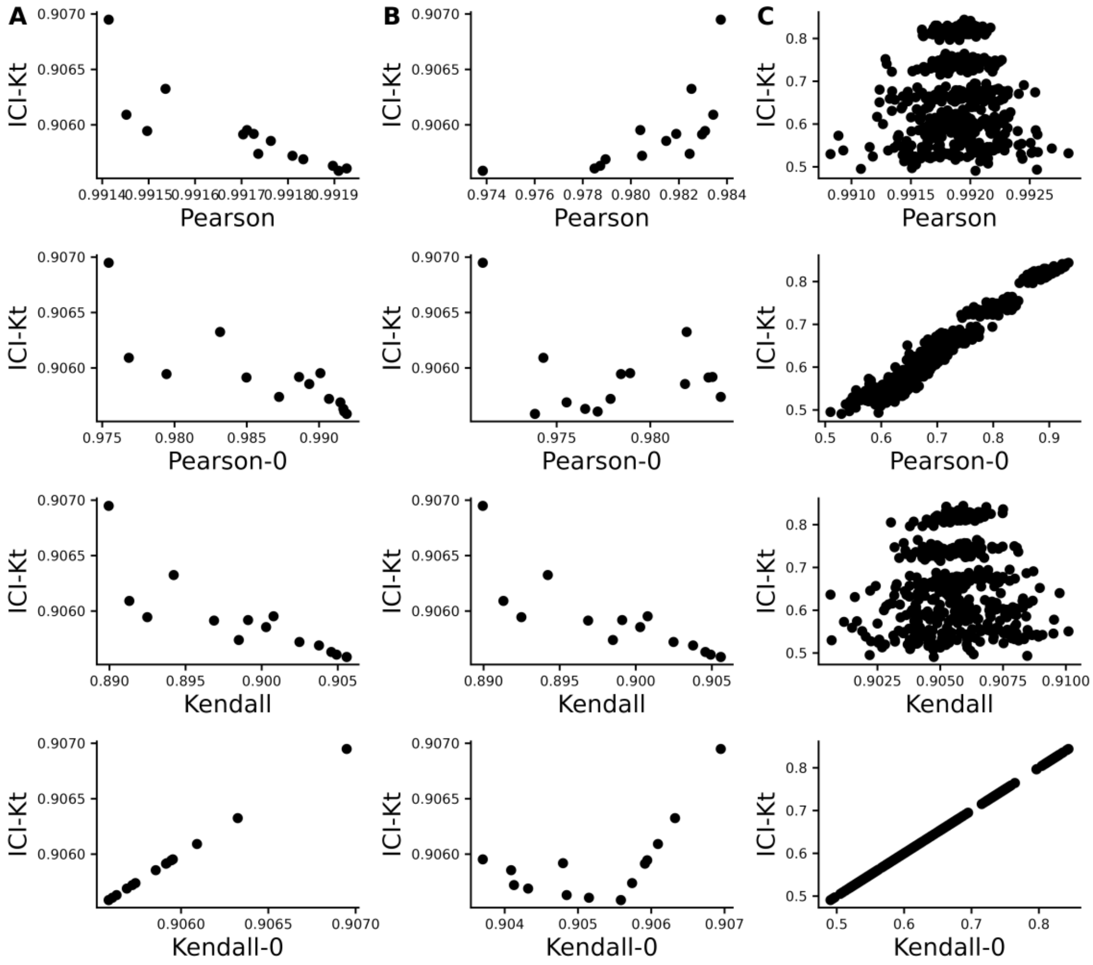

Figure S8. Effect of introducing missing values from a cutoff (A & B) or randomly (C) on different measures of correlation, including ICI-Kt, Kendall with pairwise complete, Kendall replacing missing with 0, Pearson with pairwise complete, and Pearson replacing missing with 0. A) Missing values introduced by setting an increasing cutoff. B) Missing values introduced by setting an increasing cutoff, and then log-transforming the values before calculating correlation. C) Missing values introduced at random. For the random case, each sample of random positions was repeated 100 times.

Changes In Correlation Due to Changes in Dynamic Range and Imputation

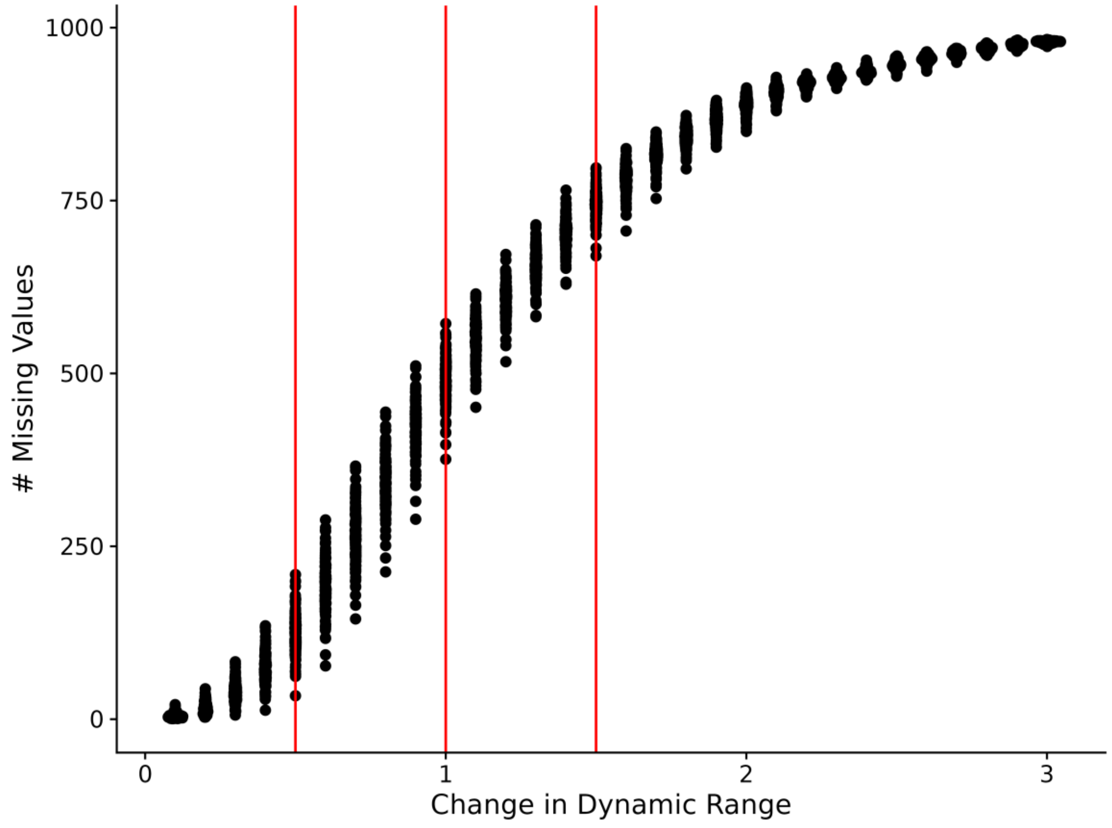

Figure S9. Number of missing values in each of the 100 samples as a function of changing the dynamic range of the values in the sample by increasing the lower limit of detection.

Outlier Detection

Table S1. The statistical results of the pairwise comparisons of each method based on the significant fractions after removing outliers. P-values were adjusted using the Bonferroni method.

Comparison	Difference	P-Value	P-Adjusted
icikt v original	0.0137	7.9e-13	3.5e-11
icikt_complete v original	0.013	1.5e-11	6.9e-10
icikt + pearson v original	0.0105	1.5e-10	6.6e-09
icikt_complete + pearson v original	0.0103	5.0e-10	2.2e-08
kt_base v original	0.0104	1.1e-09	4.9e-08

Comparison	Difference	P-Value	P-Adjusted
pearson_log1p v original	0.011	2.7e-09	1.2e-07
pearson_log v original	0.00964	4.1e-09	1.8e-07
icikt v pearson_base	0.00819	3.8e-08	1.7e-06
icikt v pearson_base_nozero	0.00949	5.5e-08	2.5e-06
icikt_complete v pearson_base_nozero	0.00888	1.5e-06	6.8e-05
icikt_complete v pearson_base	0.00758	3.6e-06	1.6e-04
pearson_base_nozero v kt_base	-0.00625	2.7e-05	1.2e-03
pearson_base_nozero v icikt + pearson	-0.00629	6.1e-05	2.7e-03
pearson_base_nozero v pearson_log1p	-0.00687	1.0e-04	4.7e-03
pearson_base_nozero v icikt_complete + pearson	-0.00618	1.4e-04	6.5e-03
pearson_base v icikt + pearson	-0.00499	1.6e-04	7.1e-03
pearson_base_nozero v pearson_log	-0.00547	2.0e-04	9.0e-03
pearson_base v kt_base	-0.00495	2.3e-04	1.0e-02
pearson_base v original	0.00547	2.6e-04	1.2e-02
pearson_base v pearson_log1p	-0.00558	2.8e-04	1.3e-02
pearson_base v icikt_complete + pearson	-0.00488	5.0e-04	2.2e-02
icikt v icikt + pearson	0.0032	5.1e-04	2.3e-02
icikt_complete v icikt_complete + pearson	0.0027	5.2e-04	2.3e-02
pearson_base_nozero v original	0.00417	1.4e-03	6.4e-02
pearson_base v pearson_log	-0.00417	1.6e-03	7.0e-02
icikt v icikt_complete + pearson	0.00331	2.0e-03	9.1e-02
icikt v pearson_log	0.00402	4.5e-03	2.0e-01
icikt v kt_base	0.00324	7.4e-03	3.3e-01
icikt_complete v pearson_log	0.00341	1.5e-02	6.7e-01
icikt_complete v kt_base	0.00263	6.6e-02	1.0e+00
icikt v pearson_log1p	0.00262	3.3e-02	1.0e+00

Comparison	Difference	P-Value	P-Adjusted
icikt_complete v icikt + pearson	0.00259	2.6e-02	1.0e+00
icikt_complete v pearson_log1p	0.002	1.6e-01	1.0e+00
pearson_log1p v pearson_log	0.0014	3.5e-01	1.0e+00
pearson_base v pearson_base_nozero	0.0013	1.6e-01	1.0e+00
pearson_log1p v icikt_complete + pearson	0.000694	5.1e-01	1.0e+00
pearson_log1p v kt_base	0.000626	6.4e-01	1.0e+00
icikt v icikt_complete	0.000613	5.5e-01	1.0e+00
pearson_log1p v icikt + pearson	0.000585	4.7e-01	1.0e+00
icikt + pearson v icikt_complete + pearson	0.00011	8.8e-01	1.0e+00
kt_base v icikt_complete + pearson	6.83e-05	9.6e-01	1.0e+00
kt_base v icikt + pearson	-4.14e-05	9.7e-01	1.0e+00
pearson_log v icikt_complete + pearson	-0.000708	5.7e-01	1.0e+00
pearson_log v kt_base	-0.000776	4.8e-01	1.0e+00
pearson_log v icikt + pearson	-0.000818	5.2e-01	1.0e+00

Feature-Feature Network Partitioning

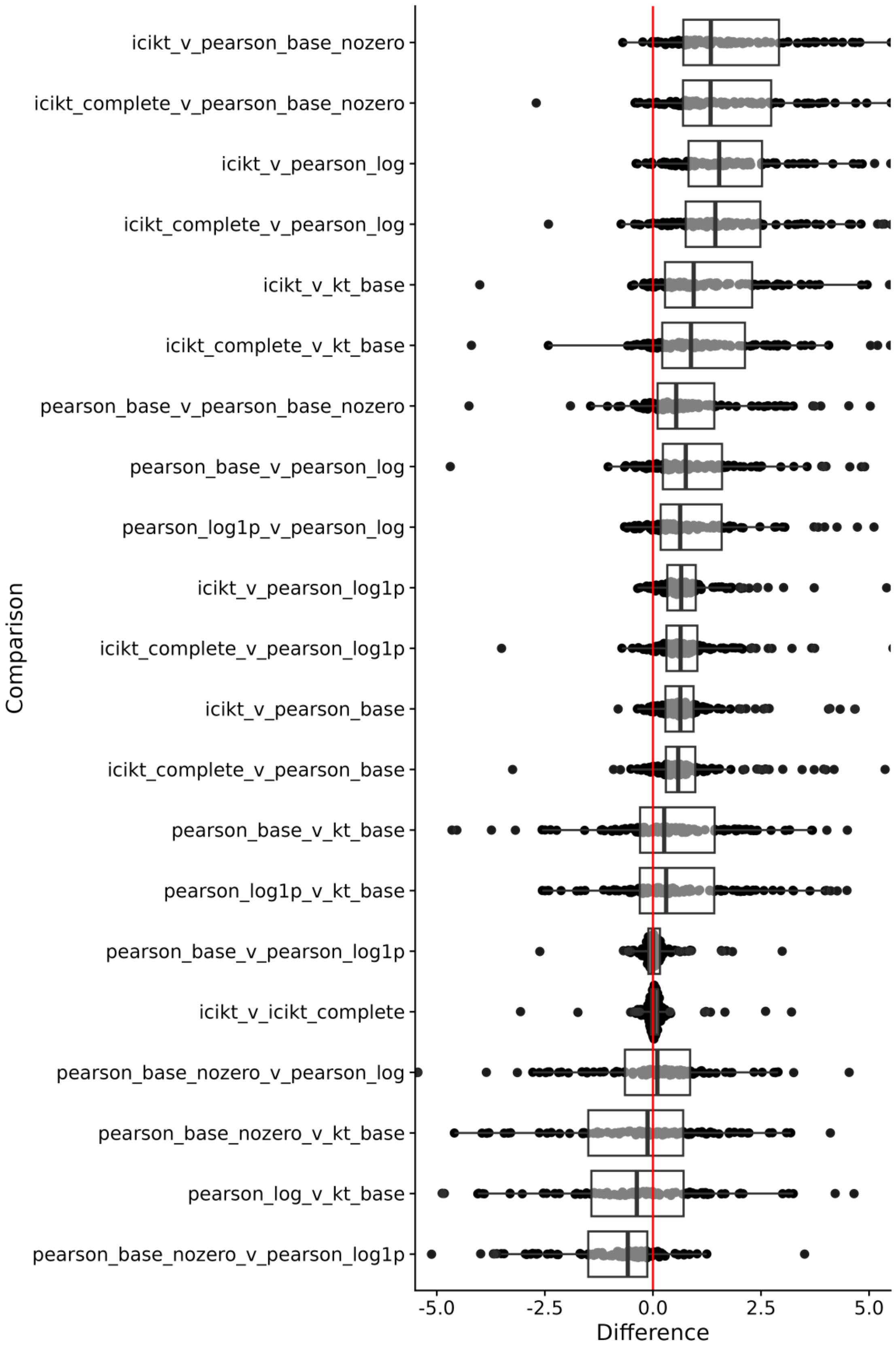

icikt_v_pearson_base_nozero
icikt_complete_v_pearson_base_nozero
icikt_v_pearson_log
icikt_complete_v_pearson_log
icikt_v_kt_base
icikt_complete_v_kt_base
pearson_base_v_pearson_base_nozero
pearson_base_v_pearson_log
pearson_log1p_v_pearson_log
icikt_v_pearson_log1p
icikt_complete_v_pearson_log1p
icikt_v_pearson_base
icikt_complete_v_pearson_base
pearson_base_v_kt_base
pearson_log1p_v_kt_base
pearson_base_v_pearson_log1p
icikt_v_icikt_complete
pearson_base_nozero_v_pearson_log
pearson_base_nozero_v_kt_base
pearson_log_v_kt_base
pearson_base_nozero_v_pearson_log1p
Comparison
-5.0
-2.5
0.0
2.5
5.0
Difference

Figure S10. Boxplot and sina plots of paired differences of partitioning ratios across datasets. Red line indicates zero difference. Differences are calculated as $method_1 - method_2$.

Table S2. Paired t-test statistics comparing all the methods to each other. P-values were adjusted using the Bonferroni adjustment.

Comparison	Difference	P-Value	P-Adjusted
icikt_complete v pearson_log	2.3	2.7e-12	5.7e-11
icikt v pearson_log	2.35	3.9e-12	8.2e-11
icikt v pearson_base_nozero	2.51	4.1e-12	8.6e-11
icikt_complete v pearson_base_nozero	2.45	7.5e-12	1.6e-10
icikt v kt_base	1.7	1.2e-10	2.6e-09
icikt_complete v kt_base	1.63	3.7e-10	7.7e-09
icikt v pearson_log1p	1.15	8.6e-09	1.8e-07
icikt_complete v pearson_log1p	1.08	9.2e-09	1.9e-07
icikt v pearson_base	1.05	6.6e-08	1.4e-06
icikt_complete v pearson_base	0.976	6.8e-08	1.4e-06
pearson_base v pearson_base_nozero	1.41	7.7e-07	1.6e-05
pearson_base_nozero v pearson_log1p	-1.33	1.7e-06	3.7e-05
pearson_base v pearson_log	1.31	3.1e-05	6.6e-04
pearson_log1p v pearson_log	1.18	3.4e-04	7.1e-03
pearson_base v kt_base	0.62	6.5e-04	1.4e-02
pearson_log1p v kt_base	0.534	1.0e-02	2.2e-01
pearson_base_nozero v kt_base	-0.668	1.9e-02	4.0e-01
pearson_log v kt_base	-0.676	4.4e-02	9.3e-01
pearson_base v pearson_log1p	0.0957	2.9e-01	1.0e+00
icikt v icikt_complete	0.0696	1.4e-01	1.0e+00
pearson_base_nozero v pearson_log	-0.196	5.6e-01	1.0e+00

Performance

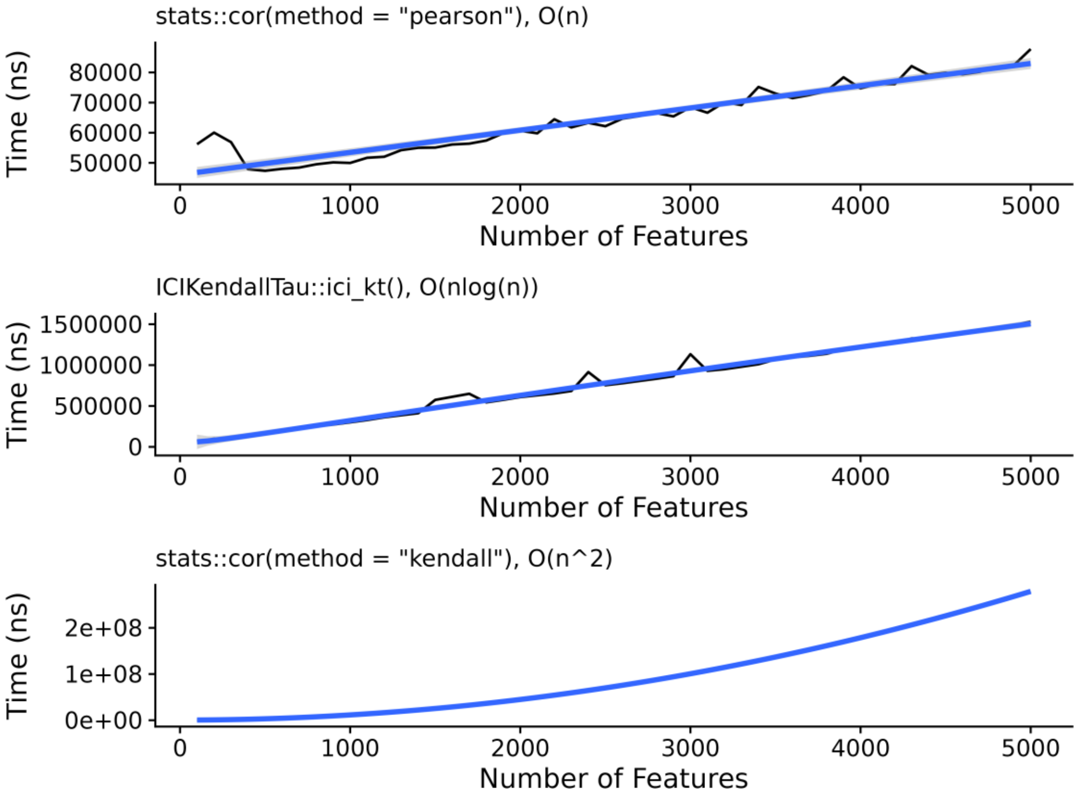

Figure S11. Time in seconds needed as a function of the number of features, with a fitted line for the assumed complexity for each of the methods tested, including R's Pearson correlation, the ICI-Kt mergesort, and R's Kendall-tau correlation algorithm.

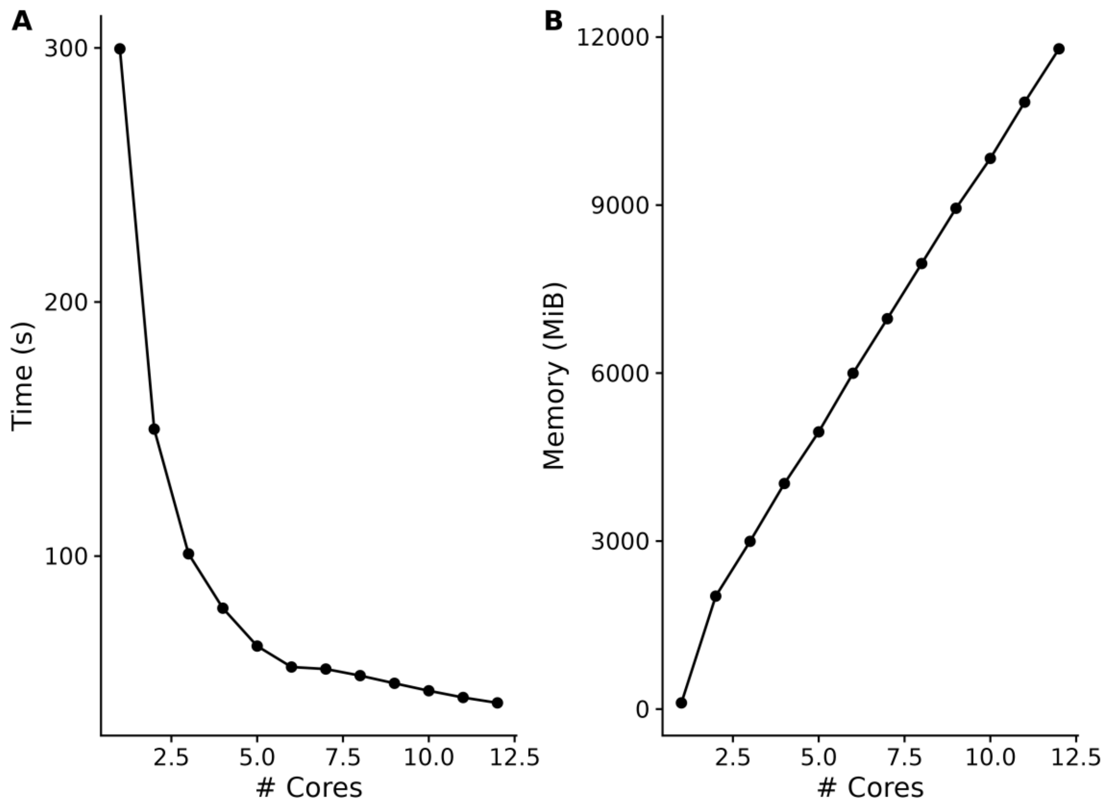

Figure S12. **(A)**: Time in seconds as the number of compute cores is increased. **(B)**: Additional memory used as the number of compute cores is increased.
